# Integrating the Theory of Planned Behaviour and Theory of Consumption Values to Explain Purchase Intentions for Stress-Reducing Beverages: A Cross-Cultural Study of New Zealand, India, and Malaysia

**DOI:** 10.3390/foods15213781

**Published:** 2026-10-22

**Authors:** Stephanie Wagner, Kevin Kantono, Sze Ying Leong, Diksha Chadha, Jipsa Ann Philip, Rothman Kam, Wee Leong Chang, Nazimah Hamid

**Affiliations:** 1 Future Foods, and Health Research Centre, School of Science, Auckland University of Technology, Private Bag 92006, Auckland 1142, New Zealandrothman.kam@aut.ac.nz (R.K.); wee.leon.chang@aut.ac.nz (W.L.C.); 2 School of Biosciences, Taylor’s University, Subang Jaya 47500, Selangor, Malaysia; szeying.leong@taylors.edu.my; 3 School of Medical and Health Sciences, Edith Cowan University, Perth, WA 6027, Australia; d.chadha@ecu.edu.au

**Keywords:** stress-reducing beverages, functional foods, theory of planned behaviour, theory of consumption values, purchase intention, partial least squares path modelling

## Abstract

Stress-reducing beverages represent a growing segment of the functional beverage market. Stress is a particularly salient concern among young consumers, who are also the category’s primary early adopters, making them the key target segment for functional beverage innovation. However, the value-based drivers of consumer adoption across different cultures lack sufficient research. This study integrated the Theory of Planned Behaviour (TPB) and the Theory of Consumption Values (TCV) to evaluate purchase intentions among predominantly young adult consumers in New Zealand, India, and Malaysia. Data from 660 participants were analysed using Partial Least Squares Path Modelling (PLS-PM) and multigroup analysis. Findings revealed that while attitudes and subjective norms predicted intention across all groups, the underlying value drivers varied by country. Functional value, specifically perceived health benefits and product safety, was the strongest driver of behavioural intention in India. Initial trust and conditional value showed the strongest associations with behavioural intention in Malaysia. In New Zealand, conditional value (promotional sensitivity) and perceived behavioural control (personal autonomy) showed stronger associations with behavioural intention than functional or social value. For industry, these results indicate that market penetration across the Asia-Pacific region requires culturally tailored rather than standardised strategies: evidence-based efficacy claims in India, certification-led trust-building and low-risk trials in Malaysia, and convenience, retail availability and price promotions in New Zealand. Because demographic composition differed across the three national samples, country-level differences should be interpreted as differences between sampled cohorts rather than established cultural effects.

## 1. Introduction

Stress can reduce the quality of life as it can compromise psychological and physiological well-being. High workloads, urbanisation, and social pressures define modern lifestyles, driving an increase in stress-related disorders across all age cohorts [1]. Young consumers, particularly students and early-career professionals, report disproportionately high stress levels linked to academic pressure, career uncertainty, and social media use, making this cohort a key demographic for stress-management product adoption [2]. Young consumers are also the commercial engine of the category. In the United States, sales of adaptogen beverages exceeded USD 1.6 billion in the year ending February 2026, and Gen Z and Millennials were the most likely to have recently consumed a non-alcoholic functional wellness drink [3]. In the Asia-Pacific region, over a third of Chinese Gen Z consumers report choosing more foods and beverages to improve their health [4]. Understanding what drives this segment’s purchase intentions is therefore central to the category’s commercial success. Functional foods and beverages offer health benefits beyond basic nutrition. Demand for these products is expanding. Plant-based probiotic beverages align with this market shift. They cater directly to populations managing lactose intolerance, milk protein allergies, high cholesterol, elevated BMI, and vegan dietary requirements [5].

Stress-relieving beverages form a functional drink sub-category [6,7]. Stress-reducing drinks are defined as non-alcoholic functional beverages formulated with calming ingredients like adaptogens, amino acids and herbs that contribute to relaxation and calmness [8]. The global kombucha market was USD 510 million in 2024 and is projected to reach USD 1.94 billion by 2033, showing a 15.2% CAGR [9]. Other projections estimate this beverage segment at USD 1654.13 million by 2032 [6]. Ashwagandha efficacy has been investigated using serum and saliva cortisol assessments over varied durations [10,11,12,13,14,15,16,17]. These studies demonstrated significant cortisol improvements. Stressed adults showed serum cortisol reductions after 14 days [15] and 30 days [16]. Manufacturers are responding by incorporating adaptogens and botanicals such as ashwagandha, L-theanine and chamomile into ready-to-drink formats, reflecting growing consumer demand for products that support emotional wellbeing [18].

However, cross-cultural consumer behaviour research on stress-relieving beverages remains limited. The value perceptions driving market adoption require further baseline investigation. For manufacturers, this gap means there is little evidence on whether a single positioning strategy can succeed across countries, or which product attributes should be prioritised in each market.

The global functional food and beverage market reached USD 162 billion in 2018, exceeding organic food retail sales of USD 115.3 billion [19]. Acceptance depends on perceived health benefits, convenience, safety, sensory attributes, and self-efficacy [20,21]. The Asia-Pacific functional beverage markets are projected to expand from USD 107.37 billion in 2025 to USD 161.89 billion by 2030 [22]. This growth varies by country. New Zealand’s natural health sector increased by 13% annually, where 80% of consumers were searching for dietary health benefits, with 37% using daily supplements for stress and sleep [23]. The Indian market was valued at USD 6.20 billion in 2024. It is projected to reach USD 16.25 billion by 2033 at an 11.3% CAGR [24], with stress-relief and adaptogen drinks supported by Ayurvedic traditions and rising urban wellness consumption [25]. Malaysia has an 8.9% projected CAGR by 2027 [26]. The consumption of functional products was primarily for immunity and digestion to reduce lifestyle diseases like obesity and hypertension.

Research on stress-reducing beverages remains limited, and while cultural norms and values are known to shape purchase intentions across different countries, the Theory of Planned Behaviour (TPB), which predicts food-related behavioural intentions through attitudes, subjective norms, and perceived behavioural control [27,28], lacks mechanisms to capture personal values and trust.

This study integrates TPB with the Theory of Consumption Values (TCV). TPB has been used to predict energy drink consumption [29]. Additional constructs can be further used to improve explanatory power [30,31,32]. However, these extensions have typically added single constructs in isolation and have rarely been tested in the context of functional/stress-reducing beverages or across culturally distinct markets. The present study’s contribution lies in integrating a multi-dimensional values framework (TCV), capturing functional, social, conditional, epistemic, and trust-related factors simultaneously, rather than a single additional construct, and in testing this integrated model comparatively across three culturally and economically distinct markets. Combining TPB with TCV allows this study to capture both the psychosocial determinants of intention central to TPB and the value-driven determinants central to TCV, offering a more comprehensive account of purchase intention than either framework alone. The specific TPB and TCV constructs examined here were selected for their established relevance to functional beverage consumption. Within TCV, functional, social, conditional, and epistemic values were selected to capture the utilitarian, social, situational, and knowledge-seeking dimensions relevant to an emerging product category. Initial trust replaced emotional value from the original TCV framework [33], as consumer evaluation of a novel, health-oriented beverage category was expected to depend more on the credibility of health-related claims than on affective response [34,35,36,37]. 

**Figure 1:**
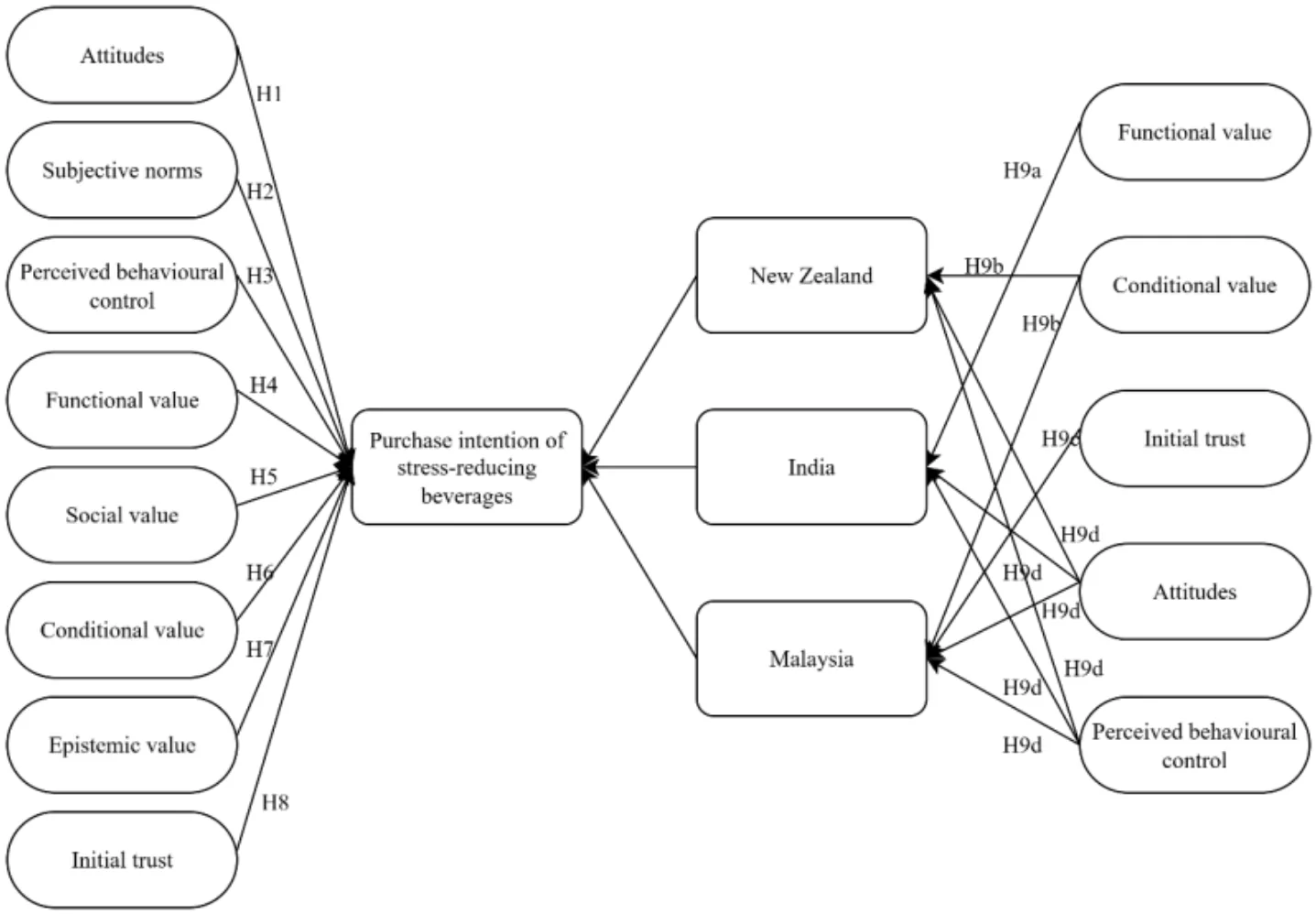
Conceptual model developed for this study.

This combined model can help assess determinants of purchase intention in New Zealand, India, and Malaysia as these countries vary in economic development, urbanisation, work-life balance, and healthcare practices. Cultural theory suggests that individualistic and collectivist societies differ in how consumers weigh social influence, personal attitudes, and tangible product benefits when forming purchase decisions [38]. As New Zealand, India, and Malaysia occupy distinct positions on this spectrum, alongside differing market maturity, the relative importance of the TPB and TCV constructs examined here is expected to vary across these contexts, motivating the country-specific hypotheses (H9a–H9d) proposed below. Building on this theoretical synthesis of contrasting market maturities to inform culture-specific marketing strategies, the following hypotheses examine the interaction between behavioural intention and value-driven factors, with the proposed relationships and integrated theoretical model illustrated in Figure 1. This figure shows the integrated TPB–TCV model: eight constructs (Attitudes, Subjective Norms, Perceived Behavioural Control, Functional Value, Social Value, Conditional Value, Epistemic Value, Initial Trust) are each hypothesised to predict Purchase Intention (H1–H8). The right side extends this by testing whether these relationships differ across New Zealand, India, and Malaysia (H9a–H9d), reflecting the study’s cross-country multigroup comparison.

The Theory of Planned Behaviour (TPB) states that a consumer’s internal mindset and social environment are primary cognitive catalysts for action. First, when consumers believe a stress-reducing beverage will provide tangible psychological relief and a pleasant sensory experience, they form a positive attitude, which acts as the foundational driver for purchase intent [33,34]. Second, dietary habits are often shaped by peer validation; if family and friends endorse the consumption of functional beverages, the individual experiences normative pressure to align their purchase behaviour with group expectations [33,35]. Finally, if consumers perceive they have the financial resources, market accessibility, and autonomy to easily acquire these beverages, this self-efficacy translates directly into a higher likelihood of purchase [36,37]. Therefore, the following hypotheses are proposed:

**H1:**  *Attitudes towards consumption of stress-reducing beverages significantly influence purchase intention of stress-reducing beverages [39,40].*

**H2:**  *Subjective norms towards consumption of stress-reducing drinks significantly influence purchase intention of stress-reducing beverages [39,41].*

**H3:**  *Perceived behavioural control of consumption of stress-reducing beverages significantly influences purchase intention of stress-reducing drinks [42,43].*

In addition to internal behavioural drivers, the Theory of Consumption Values (TCV) outlines the specific product utilities that drive market adoption. Consumers evaluating health-oriented products prioritise physiological efficacy and ingredient safety; therefore, stronger perceived functional benefits directly elevate purchase readiness [38,39]. Furthermore, consuming novel functional foods can signal health-conscious status to others, meaning anticipated social admiration can motivate acquisition [40,41]. Because functional beverages often carry premium prices and are consumed in specific contexts (e.g., high-stress workdays), promotional discounts or situational triggers (conditional value) lower barriers and stimulate immediate purchase intention [28,39]. Additionally, consumers driven by curiosity and a desire for new sensory experiences (epistemic value) are more likely to explore and purchase emerging stress-reducing variants [39,40]. Finally, in a market saturated with health claims, consumers must believe in the brand’s authenticity to mitigate perceived risk, making initial trust a prerequisite for adoption [42]. Therefore, the following hypotheses are proposed:

**H4:**  *Functional value towards consumption of stress-reducing beverages significantly influences purchase intention of stress-reducing beverages [33,44].*

**H5:**  *Social value towards consumption of stress-reducing beverages significantly influences purchase intention of stress-reducing beverages [45,46].*

**H6:**  *Conditional value towards consumption of stress-reducing drinks significantly influences purchase intention of stress-reducing beverages [34,44].*

**H7:**  *Epistemic value towards consumption of stress-reducing beverages significantly influences purchase intention of stress-reducing beverages [44,45].*

**H8:**  *Initial trust towards consumption of stress-reducing drinks significantly influences purchase intention of stress-reducing beverages [47].*

In the conceptual model, the direct relationships between these core TPB and TCV constructs and purchase intention were examined. However, based on established behavioural literature, we also evaluated the indirect pathways, specifically how subjective norms mediate purchase intention by first shaping an individual’s internal attitudes and perceived behavioural control. Furthermore, the relative strength of these behavioural drivers is expected to vary by cultural context. Cultural theory suggests that individualistic societies (e.g., New Zealand) prioritise tangible benefits and situational convenience [25,32]. Conversely, collectivist societies (e.g., India and Malaysia) rely more heavily on social norms, brand trust, and established product efficacy when navigating health decisions [17,32,43]. Building on this framework, the following hypotheses were developed:

**H9a:**  *Functional Value will exert a stronger effect on purchase intention towards stress-reducing drinks in India than in New Zealand or Malaysia [21,38].*

**H9b:**  *Conditional Value will exert a stronger effect on purchase intention of stress-reducing beverages in New Zealand and Malaysia than in India [30,38].*

**H9c:**  *Initial Trust will exert a stronger effect on purchase intention of stress-reducing beverages in Malaysia than in India or New Zealand [48].*

**H9d:**  *Attitude and Perceived Behavioural Control will consistently predict purchase intention of stress-reducing beverages across all three cohorts, while Subjective Norms will exert a stronger influence in India and Malaysia than in New Zealand [27,38].*

New Zealand, India, and Malaysia were selected because they occupy distinct positions on individualism–collectivism, economic development, and market maturity for functional beverages, providing an appropriate basis for testing H9a–H9d. India’s collectivist orientation and strong tradition of health-oriented natural remedies support functional value exerting a stronger effect on purchase intention than in New Zealand or Malaysia (H9a). New Zealand’s individualistic, more mature market and Malaysia’s intermediate market position support conditional value mattering more in both relative to India (H9b), while in Malaysia’s still-developing market, consumers can rely neither on the product familiarity of New Zealand’s more mature market nor on India’s long tradition of natural health remedies, which supports initial trust exerting a stronger effect than in India or New Zealand (H9c). As core TPB constructs, attitudes and perceived behavioural control are expected to consistently predict intention across all three cohorts, while New Zealand’s individualism is expected to reduce the influence of subjective norms relative to the more collectivist India and Malaysia (H9d).

This study applies an integrated TPB–TCV framework to evaluate the behavioural and value-based determinants of purchase intention for stress-reducing beverages across these three nations. Beyond addressing existing literature gaps, the study offers practical guidance for beverage marketers by identifying which drivers matter in each market, and therefore where a standardised strategy is likely to fail and what should replace it. In India, efficacy-led positioning builds on the established link between health consciousness and the purchase of traditional Ayurvedic products [49]. In Malaysia, trust-building through recognised certification addresses a market in which certification is a primary signal of product confidence [50]. In New Zealand, convenience- and promotion-led strategies suit a retail environment in which nearly half of household grocery purchases are made on promotion [51]. Because the study focuses on young adults, the category’s primary early adopters [52], these insights target the segment most likely to shape the category’s future, and they also inform health promotion strategies within the Asia-Pacific region.

## 2. Materials and Methods

### 2.1. Study Design and Ethics

To evaluate consumer purchase behaviour and intention toward stress-reducing beverages, a cross-sectional online survey administered using Qualtrics (Qualtrics, Provo, UT, USA) assessed target participants in New Zealand, India, and Malaysia. Between 1 May and 31 August 2025, data collection proceeded. Participants received no monetary incentives. Prior to data collection, an information sheet detailed the study objectives, participant rights, and provided a definition of stress-reducing beverages to ensure baseline comprehension.

Relevant ethics committees approved the study. Approval No. 24/142 applied to the New Zealand and India participants. In Malaysia, a separate ethics approval (Approval No. HEC2024/430) was obtained. An information sheet that detailed the study objectives and participant rights was provided. Respondents provided written electronic consent prior to data collection. Participants withdrawal was permitted at any stage with no penalty. Data were anonymised. The final datasets were saved on secure, password-protected servers. A total of 875 individuals accessed the online survey (290 in New Zealand, 305 in India and 280 in Malaysia). The survey employed forced-response settings, ensuring only fully completed questionnaires could be submitted. Therefore 157 respondents who started but did not complete the survey were not included in the dataset. Duplicate responses were screened by enabling the “Prevent multiple submissions” function in Qualtrics and checking IP addresses, which led to the removal of 19 duplicate responses. A further 39 responses were excluded because they failed the attention-check item. Recruitment in each country was closed once a quota of 220 valid responses had been reached, resulting in a final sample of 660 valid responses (220 each from New Zealand, India and Malaysia). To address concerns regarding demographic imbalance within the sampled cohorts, demographic variables were included as covariates in the PLS-PM model and their paths to purchase intention examined. This covariate-adjusted sensitivity (see Table S7) analysis indicated that demographic variables did not exert a statistically significant effect on purchase intention, with two exceptions: ethnicity showed a significant negative effect within the Indian subsample (β = −0.090, *p* = 0.022), and educational qualification showed a significant positive effect within the Malaysian subsample (β = 0.115, *p* = 0.026). No other demographic covariates, including age and gender across all three countries and ethnicity within the New Zealand and Malaysian subsamples, reached statistical significance (all *p* > 0.05). This finding is interpreted as a characteristic of the sampled Indian cohort rather than evidence of a broader cultural effect, given the non-probability, convenience-based recruitment strategy employed.

### 2.2. Participants and Sampling

Participants included adults aged 18 to 65 years from New Zealand, India, and Malaysia, recruited using non-probability convenience sampling by geographically targeted Facebook and Instagram advertisements. To ensure responses were restricted to the examined countries, advertising visibility was strictly limited to users residing in these locations. The final sample totalled 660 respondents, with an equal sample size across countries (*n* = 220 per group).

Sample size was determined a priori using the inverse square root method for PLS-SEM [53], which estimates the minimum required sample size based on the smallest path coefficient anticipated to be statistically significant. Assuming a minimum detectable path coefficient of 0.17, a minimum sample size of approximately 220 per group was required to achieve a statistical power of 80% at α = 0.05, which the achieved sample size satisfies [53,54].

The survey was administered in English across all three countries due to its established role in higher education, business, and public life in India and Malaysia, and its status as New Zealand’s primary language, though this choice may have skewed the sample toward more English-proficient, educated, and urban respondents.

### 2.3. Survey Instrument and Measures

The survey was carried out using Qualtrics in English across all participants in the three countries. Participants were presented with the following definition prior to completing the questionnaire: “Stress-reducing beverages are drinks formulated with ingredients (such as adaptogens, amino acids, or herbal extracts) marketed to help reduce stress or promote relaxation, distinct from caffeinated energy drinks.” This definition was displayed at the start of the questionnaire to ensure a consistent frame of reference across respondents.

A New Zealand pilot group (n = 10) pre-tested the survey. This pilot was conducted strictly for qualitative pre testing rather than statistical generalization. The clarity, technical functionality, and face validity of the survey items were evaluated, and refinements were made following the feedback. As the pilot study was conducted only with New Zealand participants, cross-country comprehension was not independently pre-tested in India or Malaysia prior to fielding. We acknowledge the lack of formal cross-country pre-testing (e.g., cognitive interviewing or back-translation checks) as a limitation. Distributed across three sections, the final questionnaire contained 32 items. Completion required approximately 15 min. To extract sociodemographic data, the initial section recorded variables that included age, gender, ethnicity, and annual income. Participants reported general health practices, providing information on supplement use and mindfulness activities.

Validated scales quantified purchase intention. Behavioural drivers were determined using the Theory of Planned Behaviour (TPB). The constructs evaluated were attitude, subjective norms, perceived behavioural control, and behavioural intention [27]. Perceived behavioural control (PBC) was operationalised using items adapted for autonomy and self-directed decision-making (e.g., “I will decide entirely for myself to drink stress-reducing drinks”), rather than items capturing resource-based control such as affordability or availability. As such, the PBC construct in this study reflects perceived personal autonomy over the decision, and should not be interpreted as establishing the absence of external affordability or availability barriers to consumption.

The Theory of Consumption Values (TCV) evaluated value-based drivers that measured functional, social, conditional, epistemic values, and initial trust constructs [33]. Item measurement used a 5-point Likert scale, ranging from 1 (Strongly Disagree) to 5 (Strongly Agree). Table 1 summarises the individual items and scales used. While the original TCV includes emotional value, this study substituted it with initial trust. Recent literature on functional and health-oriented products, such as the framework extension by Chakraborty et al. [48], indicates that brand trust and perceived authenticity are more critical determinants of purchase intention than emotional responses in this specific sector.

### 2.4. Sensitivity Analysis of the Behavioural Intention Measure

BI2 was measured on a frequency-based scale rather than a Likert agreement scale, because anticipated consumption frequency is a quantity rather than a matter of agreement. This item therefore captures a dimension of intention strength that BI1 and BI3 cannot, while all three items reflect the same underlying behavioural intention construct. To assess whether the conclusions depended on this composite measure, the structural model was re-estimated for each country with BI2 removed, and the path coefficients, their significance and the explained variance were compared with those of the original three-item model (Supplementary Table S8).

Across all three countries, no path changed direction, path coefficients changed by less than 0.06, and the explained variance in behavioural intention was virtually unchanged (New Zealand: R^2^ = 0.636 vs. 0.633; India: 0.705 vs. 0.701; Malaysia: 0.616 vs. 0.609). In Malaysia, the significance of all paths remained identical. Only three borderline changes were observed in the other two countries, each involving a path close to the 0.05 threshold where the parametric and bootstrap tests disagreed. In New Zealand, functional value reached significance on the parametric test (β = 0.126, *p* = 0.049) but not on the bootstrap confidence interval (95% CI [−0.016, 0.262]), and the bootstrap confidence interval for conditional value marginally included zero (β = 0.159, *p* = 0.013, 95% CI [−0.002, 0.323]). In India, the bootstrap confidence interval for perceived behavioural control marginally included zero (β = 0.139, *p* = 0.015, 95% CI [−0.003, 0.277]). These results indicate that the main findings were robust to the exclusion of BI2 and did not depend on the composite measure.

### 2.5. Data Analysis

Anonymised data from Qualtrics were exported to Microsoft Excel, with subsequent analysis using XLSTAT (version 2025.2.0; Lumivero, Denver, CO, USA). Models were estimated separately for each country in XLSTAT to explore relationships between latent variables (LV) and manifest variables (MV) using the PLS Path Modelling module, with a maximum of 3 iterations allowed for algorithm convergence (convergence criterion = 0.0001) and a centroid weighting scheme for internal estimation. Group comparisons across the three countries were tested using an adapted *t*-test based on bootstrap standard errors [55], with 100 bootstrap resamples per group. 95% confidence intervals for path coefficients were constructed using bootstrapping with 100 resamples, following the bias-corrected and accelerated (BCa) bootstrap method. To account for the three pairwise group comparisons conducted, *p*-values were adjusted using the Bonferroni correction.

This multivariate technique handles complex models with numerous interacting variables, and remains robust even if data deviate from normal distributions or strict sample size requirements [56,57].

A two-step algorithm was employed. The measurement model first estimated latent constructs after which path coefficients were derived from the structural model [58]. All latent constructs (attitudes, subjective norms, perceived behavioural control, behavioural intention, functional, social, conditional and epistemic value, and initial trust) were specified as reflective (Mode A). This specification follows established practice for TPB and consumption-value constructs, whose items are interchangeable manifestations of an underlying attitude, perception or belief rather than defining components of it; a change in the latent construct is therefore expected to be reflected in all of its indicators [59,60]. Accordingly, measurement quality was assessed using outer loadings (Supplementary Table S3), internal consistency reliability, convergent validity (average variance extracted, AVE) and discriminant validity (heterotrait–monotrait ratio, HTMT). Outer weights are reported in Supplementary Table S1 for completeness only and were not used to evaluate the measurement models. Dillon-Goldstein’s rho validated model reliability and internal consistency. Values > 0.7 confirmed good reliability. Convergent validity was considered adequate when outer loadings exceeded 0.70 and AVE exceeded 0.50, and discriminant validity was established when HTMT values were below 0.85 (conservative) or 0.90 (liberal) [60,61]. The Bootstrapping and Goodness-of-Fit (GoF) statistics reinforced this evaluation [62]. Because all data were collected through self-report questionnaires at a single point in time, common-method bias was assessed separately for each country using Harman’s single-factor test, in which all measurement items were entered into an unrotated principal component analysis; common-method bias is considered a concern if a single factor accounts for more than 50% of the total variance. Prior to comparing the validated models across countries, measurement invariance of composite models (MICOM) was assessed following the three-step procedure of Henseler et al. [61], as internal consistency within each country sample does not by itself establish that constructs are measured equivalently across countries. This involved evaluating configural invariance, compositional invariance, and the equality of composite mean values and variances for each construct across each pairwise country comparison (New Zealand–India, New Zealand–Malaysia, India–Malaysia). To compare the validated models, a multi-group permutation t-test was used. This allowed significant differences in TPB and TCV relationships across the New Zealand, India, and Malaysia participants to be identified [63]. While XLSTAT was the primary software used for PLS-PM estimation, SmartPLS 4 (SmartPLS GmbH, Oststeinbek, Germany) was used supplementarily to perform blindfolding and calculate the Stone-Geisser Q^2^ statistic for predictive relevance. 

## 3. Results

### 3.1. Sociodemographic Characteristics

The sociodemographic and lifestyle characteristics of the participants are summarised in Table 2.

Across the three countries, participants were mainly young adults aged between 18 and 25 years. The percentages were 64.09% in New Zealand, 50.91% in India, and 66.82% in Malaysia. It should be noted that the national samples differed in demographic composition (e.g., age, income, and education distribution; see Table 2), which should be considered when interpreting the between-country differences reported below. These differences give each cohort a distinct profile. The Malaysian cohort was the youngest and had the highest proportion of women (66.82% aged 18–25; 73.18% female). It was also predominantly single (75.91%), living with parents (51.36%), and degree-educated (62.73% held a Bachelor’s degree). The Indian cohort was comparatively older (31.36% aged 26–35), more often married (32.27%), and the most highly educated (34.09% held a Master’s degree), with around half reporting incomes between ₹3 and ₹10 lakh. The New Zealand cohort was gender-balanced, most commonly held a high school diploma (36.82%), and mostly reported annual incomes below NZ$30,000 (55.00%). Section 4.8 draws on these profiles to characterise the consumer segments to which the structural findings apply.

Gender distribution varied geographically. There was a slight male majority (52.27%) in New Zealand participants. Female participants were 73.18% in Malaysia, and 54.09% in India.

In terms of ethnicity, European (35.91%) and Asian (35.00%) identities were similar for New Zealand participants. The Indian participants comprised 65.00% Dravidian majority. Chinese (55.00%) and Malay (31.36%) ethnicities characterised the Malaysian participants. In terms of household structures, 51.36% of Malaysia, 43.64% of New Zealand and 25% of Indian participants stayed with parents. In terms of education, participants with Bachelor’s degrees were high in India (46.36%) and Malaysia (62.73%). High school diplomas (36.82%) were the main qualification for the New Zealand group.

In terms of lifestyle and wellness habits, juice was the most frequently consumed beverage across all countries, followed by energy drinks, which were more popular amongst participants in New Zealand (22.57%) compared to Malaysia (12.22%). In terms of supplement familiarity, ginger, turmeric, and fish oil were familiar across the three cohorts. Ginger familiarity was 14.78% in New Zealand, 14.59% in India, and 15.37% in Malaysia. Garlic familiarity was high in New Zealand (13.63%) and Malaysia (13.65%) but low in India (2.62%). In terms of botanical items, Indian participants were familiar with Reishi mushroom (14.99%), Holy basil/tulsi (11.50%), and Ashwagandha (9.20%). Malaysian respondents were familiar with Ginseng (13.38%) and Jujube/red dates (10.40%). Lavender familiarity was similar in New Zealand (9.03%), Malaysia (7.23%) and India (10.15%). Daily intake of supplements were high in New Zealand (27.27%) and Malaysia (25.91%). To manage stress, participants in New Zealand (55%) and India (47.73%) exercised. Malaysian participants were less engaged in exercise (25.91%). The percentage of the different supplements intake relate to its share of all selections. These metrics can help provide context in purchase intention and value perceptions variations in subsequent PLS-PM analysis.

### 3.2. Analysis of TPB Factors in the Conceptual Model

Table 3 summarises the analysis of each TPB construct, encompassing attitudes, norms, and value-based drivers. These results are based on a sample with a majority restricted to individuals aged 18–25 years and should therefore be interpreted with caution when generalising to broader age populations. This section reports descriptive distribution scores (percentages) to provide contextual background on the cohorts. Statistical significance of the differences between these cohorts is formally tested and reported in the PLS-PM and multigroup analyses in Section 3.4.

Regarding attitude, positive attitudes towards stress-reducing beverages dominated among participants in all three countries, the majority of whom were aged 18–25 years across all three countries. Most participants expected enjoyable (56.82–65.00%) and pleasant sensory experiences (61.36–67.27%) when consuming the beverage. India (64.09%) and Malaysia (59.09%) recorded higher agreement levels than New Zealand (49.09%) in terms of the beverage’s ability to improve health and manage stressful situations. These descriptive patterns are consistent with, though do not independently confirm, the TPB assumption that positive attitudes are associated with behavioural intention; the corresponding structural relationship (Attitude → Behavioural Intention) is reported separately in Table 4.

These findings support the TPB assumption; however, as the sample was predominantly young adults, this relationship should be interpreted as specific to the 18–25 age group rather than generalised across ages. Favourable attitudes enhance behavioural intention.

**Figure 2:**
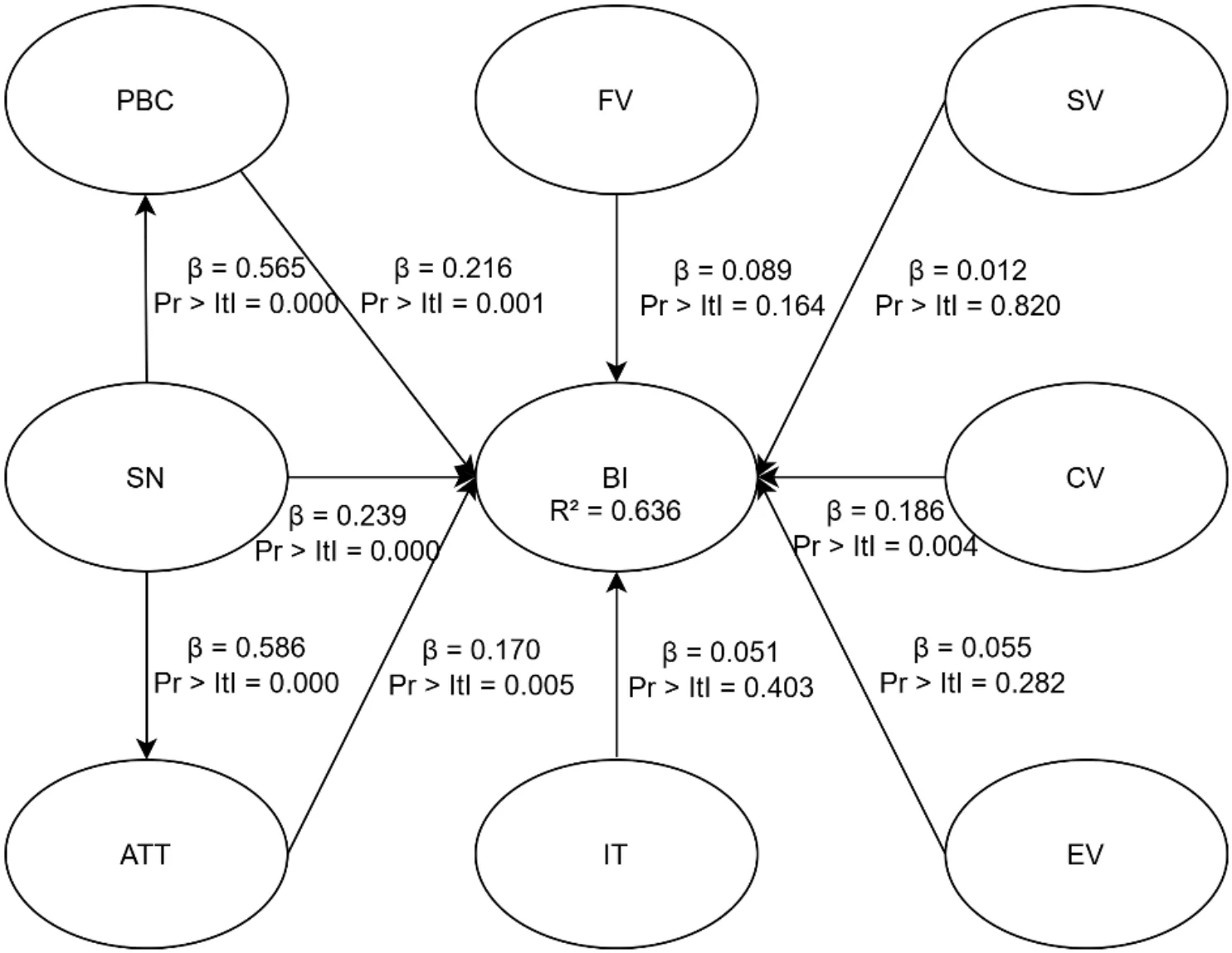
Partial least squares-path modelling (PLS-PM) results showing the path coefficients between the variables of TPB and TCV that evaluate the purchase behaviour and intention of stress-reducing drinks among consumers in New Zealand. ATT = attitude, SN = subjective norms, FV = functional value, SV = social value, CV = conditional value, EV = epistemic value, IT = initial trust, PBC = perceived behavioural control, BI = behavioural intention.

**Figure 3:**
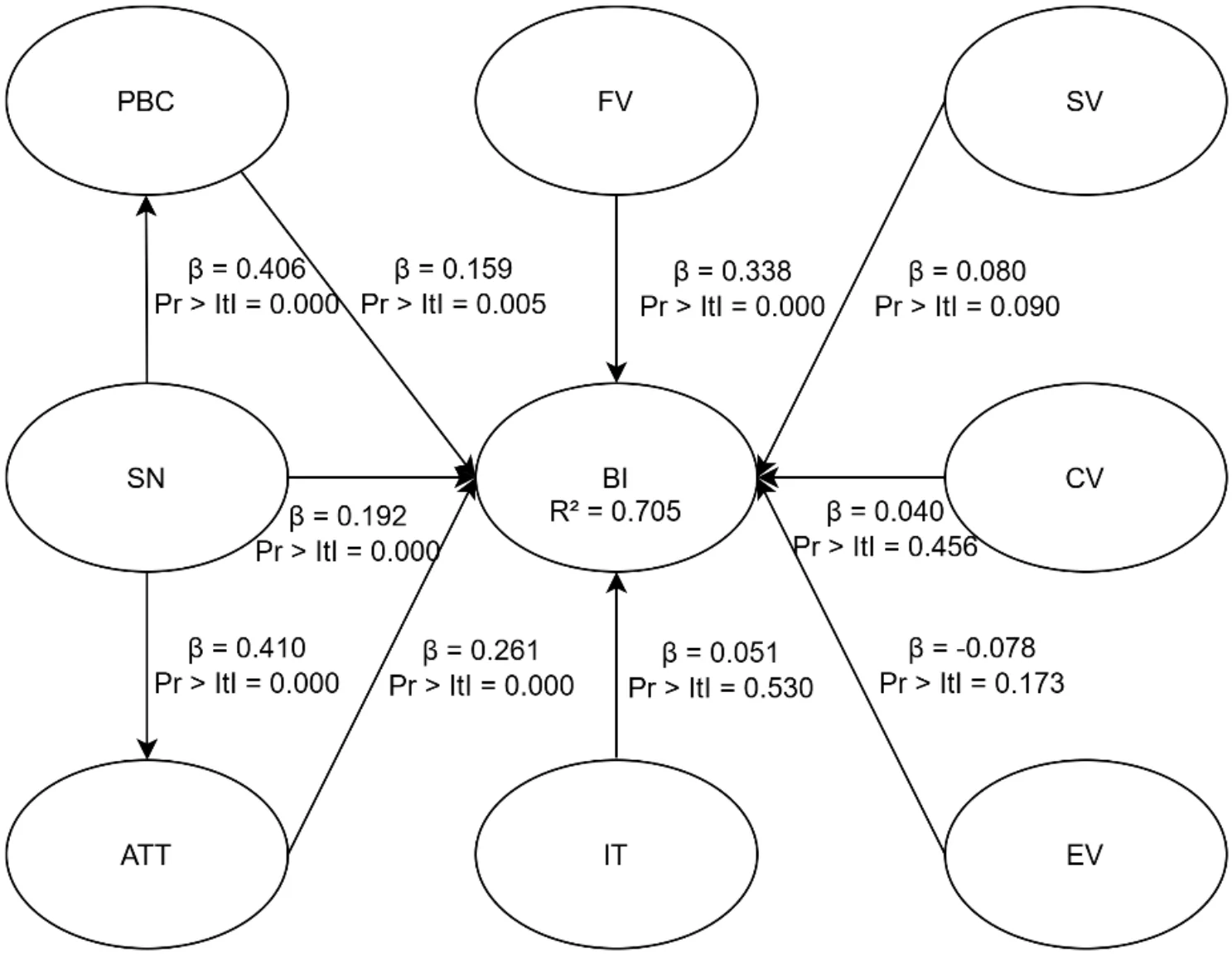
Partial least squares-path modelling (PLS-PM) results showing the path coefficients between the variables of TPB and TCV that evaluate the purchase behaviour and intention of stress-reducing drinks among consumers in India. ATT = attitude, SN = subjective norms, FV = functional value, SV = social value, CV = conditional value, EV = epistemic value, IT = initial trust, PBC = perceived behavioural control, BI = behavioural intention.

**Figure 4:**
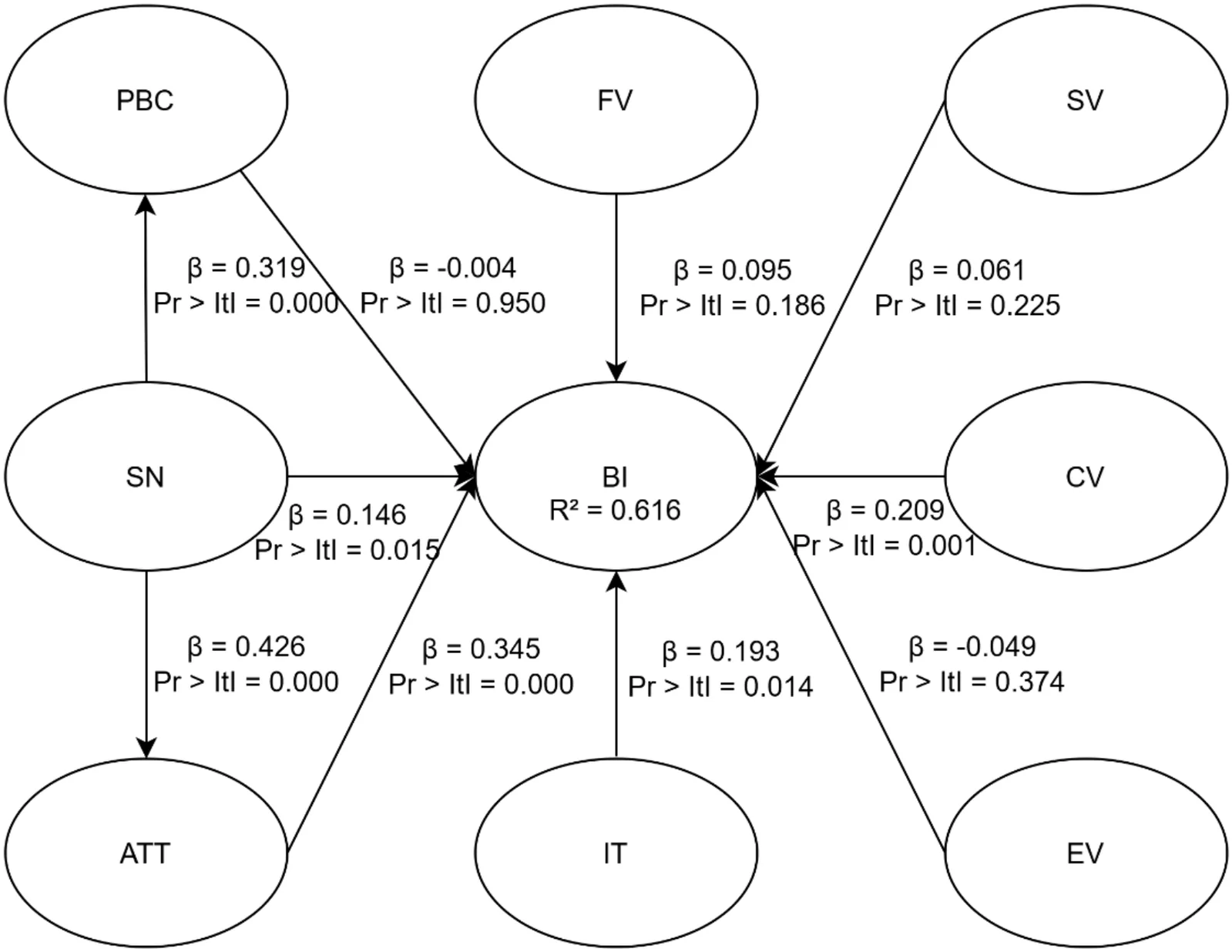
Partial least squares-path modelling (PLS-PM) results showing the path coefficients between the variables of TPB and TCV that evaluate the purchase behaviour and intention of stress-reducing drinks among consumers in Malaysia. ATT = attitude, SN = subjective norms, FV = functional value, SV = social value, CV = conditional value, EV = epistemic value, IT = initial trust, PBC = perceived behavioural control, BI = behavioural intention.

For subjective norms, the consumption of stress-reducing drinks was neither actively supported nor discouraged by friends and family. Peer consumption expectations varied across countries. Participants in New Zealand (37.73%) and Malaysia (40.91%) were more inclined to believe that their peers would consume these beverages. 42.73% of Indian participants remained neutral about this. These descriptive percentages reflect respondents’ agreement with individual survey items and do not, on their own, indicate whether subjective norms predict behavioural intention; this relationship is assessed separately via the structural path coefficient for Subjective Norms → Behavioural Intention in Figure 2, Figure 3 and Figure 4. These findings contradict the established TPB model. Social expectations did not drive the decision to buy these beverages.

In terms of perceived behavioural control, results indicate high independence across all three countries. Malaysia had the highest agreement (70.91%) regarding absolute control over the decision to intend to purchase stress-reducing beverages, followed by New Zealand (68.64%) and India (67.27%). Participants seemed to ignore external social influence. Between 48.18% and 57.73% of participants intended to consume these drinks independently. Final purchase intention decisions remained mainly with the individual participants (64.09–68.18%). Perceived controllability during the actual transaction was largely neutral (41.36–46.82%). Self-efficacy was a critical determinant of intention. These data directly confirm that specific TPB emphasis that participants feel capable of independent action. Such independence indicated that perceived barriers, around product availability or affordability, are not significant constraints to adoption.

Behavioural intention metrics varied with different countries. New Zealand participants had mainly neutral scores regarding intention of consumption of stress-reducing beverages (37.27%). India (40.91%) and Malaysia (55.91%) reported higher probability of consumption intention. Across all three countries, over 90% of participants restricted their consumption intention to three days or fewer per week, highlighting a discrepancy between general purchase readiness and planned routine consumption. Peer endorsement intentions were similar. Malaysian participants were highly inclined to recommend these products (50%). Neutral intentions characterised the New Zealand (41.82%) and Indian (44.09%) participants. Increased behavioural intention indicates progressive market readiness in Malaysia. The low consumption frequency of these beverages indicate that they have not been incorporated into daily routines and are in the early stages of market adoption. It should be noted that these findings on subjective norms, perceived behavioural control, and behavioural intention are specific to a cohort with a majority of participants aged 18–25 and, given the predominantly young-adult composition of the sample across all three countries, should not be generalised to other age groups.

### 3.3. Analysis of TCV Factors in the Conceptual Model

The functional value construct assessed the perceived utility of stress-reducing beverages concerning health efficacy and product safety. Participants across all three nations strongly associated these products with health improvement and stress reduction, with the highest levels of agreement observed in Malaysia (60.91%), followed by India (50.91%) and New Zealand (45.45%). A significant cross-cultural distinction emerged regarding product purity and safety standards. While over half of the Indian cohort (51.36%) expressed confidence that these beverages are free from additives, respondents in New Zealand remained predominantly neutral (53.18%). Similarly, Indian consumers demonstrated the highest confidence in overall product safety (47.27% agreement), whereas New Zealand (55.91%) and Malaysia (49.55%) reported high levels of neutrality. These findings suggest that while the “health-benefit promise” is widely accepted across the Asia-Pacific, tangible trust in ingredients and safety is a more prominent driver in the Indian market.

Regarding social value, constructs of TCV showed that in New Zealand (49.55%), India (38.64%) and Malaysia (43.64%) people are neutral in admiration by other people when consuming stress-reducing beverages. They neither feel accepted nor unaccepted by others when buying stress-reducing beverages in New Zealand (51.82%) and Malaysia (45.00%) whereas Indian participants strongly to somewhat disagree in feeling accepted by others when buying stress-reducing beverages (40.00%). New Zealander (50.91%), Indian (37.27%), and Malaysian (46.82%) participants neither agree nor disagree in feeling respected by others when buying stress-reducing drinks. The same number of study participants in India (37.27%) strongly or somewhat disagree feeling respected by others when buying stress-reducing beverages (37.27%). SV4 (Consuming stress-reducing beverages supports me in how people see and assess me) is rated neutral in all three countries (New Zealand 48.64%, India 39.55%, and Malaysia 43.64%).

In terms of conditional value, the analysis examined the influence of situational factors and promotional incentives on substituting conventional drinks with stress-reducing beverages. In Malaysia (67.27%) and New Zealand (52.27%), discounts and promotions were significant purchase intention triggers. The majority of Indian participants had neutral responses (58.64%) towards price-based incentives. High “situational switching” behaviour intention was evident with participants in Malaysia, with 47.73% agreeing that they would substitute conventional energy drinks for stress-reducing beverages under specific circumstances. Intrinsic health values influence Indian consumption. Price incentives influence the New Zealand and Malaysian participants. Both demographics respond to situational marketing and targeted discounts.

For epistemic value, the focus was on how curiosity and information-seeking influence adoption of the beverages. Participants in the Asian markets were highly research-driven, with 70% of Malaysians and 61.36% of Indians examining product details before buying, compared to just 42.73% in New Zealand. In addition, over 60% of participants in both Asian countries viewed trying these drinks as a “new adventure.”

Finally, initial trust examined participants’ belief in product effectiveness and authenticity. The depth of this trust varied with participants from different countries. Indian (54.09%) and Malaysian (42.27%) participants showed product trustworthiness, while New Zealand was neutral about this (40.91%). Belief in product authenticity showed a similar trend. New Zealanders were more cautious, with 46.64% being neutral, compared to the strong agreement seen in the Asian markets. This suggests that while promoting product authenticity drives sales in India and Malaysia, New Zealand consumers need more evidence before they will trust a brand. As with the preceding TPB findings, it should be noted that these functional, social, conditional, epistemic, and trust-related consumption values are drawn from a sample predominantly aged 18–25 across all three countries, and should therefore not be generalised to other age groups.

### 3.4. Incremental Explanatory Power of the Integrated TPB–TCV Model

To assess whether the TCV constructs improved the explanatory power of the TPB, a baseline model including only the core TPB constructs (attitudes, subjective norms and perceived behavioural control) predicting behavioural intention was compared with the integrated TPB–TCV model. The baseline TPB model explained 56.2% of the variance in behavioural intention (R^2^ = 0.562; Q^2^ = 0.359). Adding the TCV constructs increased the explained variance to 63.1% (R^2^ = 0.631), an increase of 6.9 percentage points (ΔR^2^ = 0.069), corresponding to a medium effect size (f^2^ = 0.187; [65]). Predictive relevance also improved substantially, with Q^2^ rising from 0.359 to 0.544 (ΔQ^2^ = 0.185). Both models showed predictive relevance (Q^2^ > 0), but the integrated model exceeded the threshold of 0.50 for large predictive relevance [60]. These results confirm that incorporating the TCV constructs considerably strengthens the explanatory and predictive power of the TPB for behavioural intention.

### 3.5. The Relationships Between TPB and TCV Using PLS-PM

Prior to evaluating the structural paths, the measurement models for all three national cohorts were assessed for reliability and internal consistency. Table 5 summarises the measurement model statistics, including Dillon-Goldstein’s rho, Cronbach’s alpha, average variance extracted (AVE), goodness-of-fit (GoF), and bootstrapping results, alongside model fit comparisons across the three national samples, to facilitate direct comparison of measurement quality and model robustness between countries.

Harman’s single-factor test indicated that the first unrotated factor explained 39.20% of the total variance in the New Zealand sample, 44.57% in the Indian sample and 36.72% in the Malaysian sample. As all values were below the 50% threshold, no single factor accounted for the majority of the variance, indicating that common-method bias was not a pervasive concern in this study [66].

#### 3.5.1. New Zealand

The PLS-PM analysis for the New Zealand participants (Figure 2) showed that four factors drove the intention to buy stress-reducing beverages. They included perceived behavioural control (β = 0.216, *p* < 0.01), conditional value (β = 0.186, *p* < 0.01), attitudes (β = 0.170, *p* < 0.01), and subjective norms (β = 0.239, *p* < 0.01). It was found that New Zealanders make purchase decisions based on social expectations, personal autonomy, and situational promotions.

The results showed a positive link between subjective norms and both attitudes (β = 0.586, *p* < 0.01) and perceived behavioural control (β = 0.565, *p* < 0.001). This suggests an overlap between personal views and perceived social pressure. The model showed high reliability, with strong internal consistency (Dillon-Goldstein’s ρ > 0.70) and a satisfactory Goodness-of-Fit (GoF = 0.546).

Analysis of indicator weights can help explain which items contributed most to the composition of each construct. Within Attitude, the highest weights were contributed by health improvement (ATT4, 0.282) and perceived importance (ATT3, 0.272). Within Conditional Value, the highest weight was contributed by participants’ reported tendency to substitute conventional drinks for stress-reducing beverages under specific environmental stressors (CV2, 0.411). Within Epistemic Value, the highest weights were contributed by information-seeking and comparison of options (EV1, 0.468; EV2, 0.487), while perceived brand trustworthiness contributed the highest weight to Initial Trust (IT2, 0.412).

These indicator weights describe how each construct was internally composed and do not, on their own, indicate predictive validity or evidence that a construct is a driver of purchase intention; the predictive relevance of TPB and TCV constructs for behavioural intention is assessed separately via the structural path coefficients reported in Table 4.

PLS-PM analysis for New Zealand, India, and Malaysia was conducted on a sample restricted to a majority of young adults aged 18–25, and caution should therefore be exercised when generalising these results to broader populations. In practical terms, this means young New Zealand consumers treat stress-reducing beverages as a situational convenience, driven by promotional discounts and personal autonomy rather than intrinsic health claims.

#### 3.5.2. India

The PLS-PM analysis for Indian participants (Figure 3) identified four significant positive predictors of purchase intention that included behavioural control (β = 0.159, *p* < 0.01), subjective norms (β = 0.192, *p* < 0.01), functional value (corr = 0.338, *p* < 0.001), and attitudes (β = 0.261, *p* < 0.001). Functional value was the strongest determinant in the model, indicating that Indian participants placed high importance on the tangible utility of stress-reducing beverages.

Subjective norms correlated strongly with both attitudes (β = 0.410, *p* < 0.001) and perceived behavioural control (β = 0.406, *p* < 0.001). This demonstrates that social expectations directly inform personal evaluations and self-efficacy among Indian participants. Model reliability was confirmed with Dillon-Goldstein’s ρ > 0.70 across all constructs, and the overall GoF was satisfactory (GoF = 0.512, bootstrapped = 0.518).

Indicator weights indicated which items contributed most to the composition of each construct. Within Attitude, the largest contributors were the belief that these drinks help manage stress (ATT5, 0.273) and improve health (ATT4, 0.241). Within the TCV framework, Functional Value was most strongly composed of perceived health improvements (FV1, 0.325), while Epistemic Value was most strongly composed of the desire for a “new adventure” (EV3, 0.475). Initial Trust was most strongly composed of perceived trustworthiness of the beverage category itself (IT2, 0.374).

These indicator weights describe how each construct was internally composed and do not, on their own, indicate that a construct predicts purchase intention; such effects are assessed separately via the structural path coefficients reported in Table 4.

These results validate the TPB framework while showing that functional value was the critical TCV driver in India. Unlike New Zealanders, young Indian participants based their purchase intentions mainly on product efficacy and health outcomes. In practical terms, this demonstrates that for this Indian demographic, the tangible health utility, efficacy, and product safety of the beverage heavily outweigh social pressure or situational discounts.

#### 3.5.3. Malaysia

The PLS-PM analysis for the Malaysian participants (Figure 4) identified four significant predictors of purchase intention: that included conditional value (β = 0.209, *p* < 0.01), initial trust (β = 0.193, *p* < 0.05), attitudes (β = 0.345, *p* < 0.001), and subjective norms (β = 0.146, *p* < 0.05). Malaysian participants were the only group where initial trust was a direct determinant, indicating the importance of brand credibility and product authenticity in this specific market.

Subjective norms were strongly correlated with both attitudes (β = 0.426, *p* < 0.001) and perceived behavioural control (β = 0.319, *p* < 0.001). This suggests that personal attitudes and self-efficacy influenced social expectations for participants in Malaysia. Model reliability was confirmed with Dillon-Goldstein’s ρ > 0.70 across all constructs, alongside a satisfactory Goodness-of-Fit (GoF = 0.607, bootstrapped = 0.618).

Indicator loadings showed the primary drivers for each construct. Attitudes were driven by the importance of consumption (ATT3, 0.277) and the ability to manage stress (ATT5, 0.274). Within the TCV framework, conditional value was primarily driven by participants’ substitution of conventional beverages during stressful circumstances (CV2, 0.446). Epistemic value was defined by information gathering and option comparison (EV2, 0.431), while initial trust was linked to the perceived trustworthiness of the overall beverage category (IT2, 0.413).

These results confirm the predictive validity of the TPB framework and identify conditional value and initial trust as the primary TCV variables for Malaysian participants. Unlike the Indian participants who prioritised functional utility, purchase intentions of Malaysian participants relied on product authenticity and situational factors. Marketing strategies for Malaysia should therefore focus on establishing credibility through certifications (e.g., Halal or transparent health labelling) and targeted promotions, rather than depending on status-based or purely health-focused marketing. In practical terms, this indicates that young Malaysian consumers, a cohort that was predominantly female and degree-educated, require strong brand authenticity and trust-building, alongside situational relevance, before committing to a purchase.

For a complete overview, Table 4 summarises the structural model results, including path coefficients (β), standard errors, t-values, *p*-values, and effect sizes (f^2^) for New Zealand, India, and Malaysia.

#### 3.5.4. Measurement Invariance of Composite Models (MICOM)

Prior to comparing structural path coefficients across countries, measurement invariance of composite models (MICOM) was assessed for each pairwise country comparison (New Zealand–India, New Zealand–Malaysia, India–Malaysia) following the three-step procedure of Henseler et al. [61]. Configural invariance (Step 1) was established across all comparisons, as identical indicators, algorithm settings, and data treatment were applied to each country sample.

Compositional invariance (Step 2) was established for all constructs across all three country pairs, with one exception: for Epistemic Value in the New Zealand–India comparison, the correlation between composite scores fell narrowly below the 5% quantile of the permutation distribution (c = 0.979, *p* = 0.030). Although this indicates a statistically detectable deviation, the correlation remains very close to unity, suggesting that the composite is formed in a highly similar, though not strictly identical, manner in both samples.

Among constructs for which compositional invariance was established, full measurement invariance—supported by equal composite mean values (Step 3a) and equal variances (Step 3b)—was found for a subset of construct–country pair combinations (e.g., Attitude and Behavioural Intention between New Zealand and India; Functional Value between New Zealand and Malaysia; Social Value across most pairs). For the majority of constructs and country pairs, however, only partial measurement invariance was established: compositional invariance held, but composite mean values and/or variances differed significantly between groups (*p* < 0.05). A full breakdown of Step 2, 3a, and 3b results for each construct across all three country pairs is reported in Supplementary Table S5.

Consistent with Henseler et al. [61], where partial invariance was established, comparisons of structural path coefficients between the relevant country pairs remain statistically valid, although comparisons of construct mean levels for these constructs should be interpreted with caution. For Epistemic Value in the New Zealand–India comparison, compositional invariance was not formally established. However, given the near-unity composite correlation (c = 0.979), the degree of non-equivalence appears small and is unlikely to substantively distort structural comparisons. Nonetheless, differences in path coefficients involving Epistemic Value between these two samples are interpreted conservatively, as they may partly reflect measurement differences rather than substantive differences between the sampled cohorts.

#### 3.5.5. Robustness to Demographic Covariates

To assess whether the country-specific structural results could be attributed to differences in the demographic composition of the cohorts, the structural model for each country was re-estimated with age, gender, ethnicity, marital status, educational qualification, income, household composition and household size included as control variables on behavioural intention. Only two covariates had a significant effect: ethnicity in the Indian subsample (β = −0.090, *p* = 0.022; f^2^ = 0.024) and educational qualification in the Malaysian subsample (β = 0.115, *p* = 0.026; f^2^ = 0.023). All other demographic covariates were non-significant in all three countries (all *p* > 0.05), and the direction and significance of the hypothesised paths remained unchanged (Supplementary Table S6). These results suggest that behavioural intention differed slightly between ethnic groups within the Indian cohort and between educational levels within the Malaysian cohort, although both effects were small. Overall, the driver patterns reported in Section 3.5.1, Section 3.5.2 and Section 3.5.3 were robust to the demographic variation within each cohort.

#### 3.5.6. Cross-Country Comparison of TPB and TCV Relationships in Stress-Reducing Beverage Purchase Behaviour

The multigroup *t*-test results (Table 6) showed significant cross-country variations in the structural paths between TPB and TCV constructs. As with the PLS-PM analysis, the multigroup *t*-test comparisons were similarly conducted on a sample restricted to a majority of young adults aged 18–25, and these results should be interpreted within that age-specific context.

##### TPB Path Variations

The influence of Subjective Norms (SN) on Perceived Behavioural Control varied significantly across the participants from New Zealand and India as well as between New Zealand and Malaysia (*p* < 0.05). Structural pathways connecting Perceived Behavioural Control to Behavioural Intention differed between India and Malaysia (*p* < 0.05).

##### TCV Path Variations

The effect of Functional Value (FV) on purchase intention differed significantly between India and New Zealand (*p* < 0.01), as well as between India and Malaysia (*p* < 0.05). The impact of Conditional Value (CV) varied significantly only between participants from India and Malaysia (*p* < 0.05).

## 4. Discussion

These findings come from cohorts in which most participants were aged 18–25. This young adult group is the category’s most commercially relevant segment: as ‘digital natives’ facing transitional life stressors such as study, career entry, and financial independence, they are the primary early adopters driving the shift toward functional, stress-reducing beverages over conventional soft drinks or alcohol [52,67]. The national samples were not demographically matched, so country-level differences should be read as differences between sampled cohorts rather than established cultural effects. However, including demographic characteristics as covariates did not significantly alter purchase intention, with two exceptions: ethnicity within the Indian subsample and educational qualification within the Malaysian subsample. The country-specific patterns therefore hold across the demographic variation within each cohort, and each national sample can be read as a description of a distinct, commercially relevant consumer segment. Section 4.8 develops these into market personas for regional marketers.

Results fully supported H1 and H2, with attitudes and subjective norms significantly predicting purchase intentions across all three countries. However, H3 was only partially supported, as perceived behavioural control significantly predicted intention in New Zealand and India, but was non-significant in Malaysia. Path coefficients for functional value (H4) and conditional value (H6) differed by country. Neither social value (H5) nor epistemic value (H7) reached significance.

The cross-cultural comparisons provided mixed support for H9a–H9d. Consistent with H9a, functional value dominated the Indian results, showing a strict health-oriented consumption pattern. Consistent with H9b, conditional value was a significant driver in Malaysia and New Zealand but not in India. New Zealand participants responded most to convenience and promotions. H9c was not supported: although initial trust significantly predicted purchase intention only in Malaysia, the multigroup analysis did not confirm that this effect was significantly stronger than in India or New Zealand. H9d was only partially supported: attitudes predicted intention consistently across all three countries, but perceived behavioural control was non-significant in Malaysia. Moreover, contrary to the expectation that individualistic cultures prioritise autonomy and transactional benefits over social influence, subjective norms also significantly predicted intention in New Zealand.

While the observed cross-country differences in functional value and conditional value are consistent with the cultural explanations proposed in H9a and H9b, an alternative explanation cannot be ruled out, because the national samples were not demographically matched. A covariate-adjusted sensitivity analysis showed that most demographic variables, including age and income, did not significantly affect purchase intention, with only small effects of ethnicity in India and educational qualification in Malaysia (Supplementary Table S6). Nevertheless, the cross-cultural differences reported here should be interpreted as descriptive and associative rather than causal, since country of residence was not experimentally manipulated and covaries with unmeasured or imperfectly controlled demographic differences between samples.

When demographic characteristics were included as covariates in the structural model, none had a significant effect on purchase intention, with two exceptions: ethnicity in the Indian subsample (β = −0.090, *p* = 0.022) and educational qualification in the Malaysian subsample (β = 0.115, *p* = 0.026) (Supplementary Table S7). This indicates that the country-specific driver patterns remain constant across demographic variations within each cohort. Hence, the distinct demographic composition of each national sample can be interpreted not only as a constraint on generalisability, but also as a description of three commercially relevant consumer segments, which Section 4.8 develops into market personas for regional marketers.

### 4.1. What Drives Purchase Intention of Stress-Reducing Beverages Across Countries?

In every market, young consumers intended to buy stress-reducing beverages when they believed the products would help them manage stress and improve their health, consistent with the core TPB assumption that favourable evaluations drive intention [27,28]. In practical terms, no marketing strategy is likely to succeed in any of these markets unless consumers are first convinced that the product works and is pleasant to consume.

Social endorsement also mattered in all three countries. This may reflect the novelty of the category: when a product type is new and its benefits are hard to judge before trying it, consumers look to friends and family to confirm that it is a sensible choice [31,32]. Many participants lived with parents or flatmates (Table 2), so purchase decisions may also be shaped by the people with whom young consumers share food and drink. The influence of social expectations was not limited to the collectivist markets, which suggests that for this age group, peer and family endorsement is a general feature of adopting new wellness products rather than a purely cultural one [68].

Feeling able to buy the product predicted intention in New Zealand and India, but not in Malaysia. One practical explanation is that Malaysian participants already reported the highest sense of control over the decision (70.91%), so control no longer distinguished between those who did and did not intend to buy. For these consumers, the question was not whether they could buy the product, but whether they could trust it, which is consistent with the role of initial trust in this market (Section 4.3).

### 4.2. Functional Value as a Primary Driver in India

For Indian participants, the most important reason to buy was the belief that the beverage delivers real health benefits and is safe to consume. Functional value did not influence intention in Malaysia or New Zealand. This aligns with evidence that health values strongly predict functional beverage intentions among young consumers [21], and with findings that health attributes carry more weight in developing markets than in Western contexts such as New Zealand [30].

This health-first orientation is understandable in its real-world context. Over 50% of Indian adults report daily, disruptive stress [69], which turns stress relief from a lifestyle choice into a practical need. It also fits with traditional Ayurvedic practice, in which herbal remedies are expected to deliver tangible effects; participants’ familiarity with ingredients such as Holy Basil/Tulsi (11.50%) and Ashwagandha (9.20%) reflects this. Because stress-reducing beverages are an emerging category in India, consumers appear to rely on concrete evidence of benefit and safety cues, such as additive-free labels, to reduce the risk of trying something new. By contrast, health claims alone were not enough to drive intention in Malaysia or New Zealand, supporting Kowalska et al. [19], who found that convenience and sensory appeal often outweigh health claims in Western and European-oriented markets.

### 4.3. Conditional Value and Initial Trust as Malaysian Purchase Drivers

Malaysian participants intended to buy when two conditions were met: they trusted that the product was genuine and effective, and it fitted the situations in which they needed it, including when it was on promotion. Initial trust did not influence intention in India or New Zealand. This aligns with Jia and Liu [40], who showed that initial trust is important when consumers evaluate health-oriented products and helps other consumption values translate into intention. In real-world terms, trust acts as a gatekeeper. Malaysia reports high stress prevalence, at over 60% [70], and a crowded market with many products and complex regulatory signals. In such an environment, consumers need reassurance that a product is authentic before committing to it [19]. Conditional value adds a situational dimension: Malaysian participants were willing to switch from conventional energy drinks during specific stressful periods, and they responded strongly to discounts. Market penetration in Malaysia therefore depends on both trust-building and situational relevance. For a young, predominantly female consumer base, these strategies should centre on reducing the perceived risk of trying a novel health product. Examples include visible third-party and regulatory certification (such as halal or Ministry of Health approval, where relevant), transparent ingredient sourcing, and endorsement by credible health professionals rather than lifestyle influencers, given that social value was non-significant. Because conditional value was also significant, trust-building can be paired with low-commitment trial formats, such as single-serve packs, sampling, or introductory discounts timed to predictable high-stress periods. Together these allow consumers to verify product authenticity before committing to repeat purchase.

### 4.4. Conditional Value and Perceived Behavioural Control as Purchase Drivers in New Zealand

New Zealand participants intended to buy stress-reducing beverages when doing so was easy, affordable, and within their control. Functional value and initial trust did not influence intention. In practice, these consumers appear to treat stress-reducing beverages as a convenient, situational purchase rather than a health intervention, consistent with Malavalli et al. [30], who reported that situational factors often outweigh health considerations in individualistic cultures, and with Treloar et al. [29], who found that perceived control and situational factors strongly influence energy drink consumption among New Zealand students. The sample’s comparatively lower incomes (Table 2) further help explain why price and promotions mattered.

Social expectations also influenced New Zealand participants, which tempers the assumption that consumers in individualistic cultures are indifferent to social influence. A practical explanation lies in their living arrangements: many participants lived with parents or flatmates, where food and drink purchases are often shared or visible to others. New Zealand consumers therefore combine a strong sense of personal agency with sensitivity to the views of those around them. While Indian participants prioritise functional efficacy and Malaysian participants rely on trust, New Zealand consumers are driven by personal control, social endorsement, convenience, and financial incentives.

### 4.5. The Non-Significant Influence of Social and Epistemic Values

Across all three markets, practical considerations such as efficacy in India, trust in Malaysia, and convenience in New Zealand mattered more than social recognition.

This aligns with Chadha et al. [67], who observed that epistemic value plays a minor role in dietary choices when functional benefits are clearly communicated. One possible explanation is the internal nature of stress: because stress relief is experienced privately, consumers may prioritise whether a product works over its novelty or image, although this interpretation was not directly tested in the present model. For marketers, this suggests that status-oriented or influencer-led positioning is unlikely to be effective for this category.

### 4.6. Discrepancies in Planned Consumption Frequency

While Indian and Malaysian participants reported comparatively high purchase intention, planned consumption frequency (BI2) was low across all three countries. As both measures reflect prospective intention rather than observed behaviour, and no subsequent behavioural data were collected in this study, this pattern should not be interpreted as evidence of a gap between intention and actual behaviour, nor as indicating barriers to habit formation. Occasional planned use is not inconsistent with positive purchase intention; consumers may intend to buy stress-reducing beverages while expecting to use them situationally, for example during periods of elevated stress, rather than as part of a fixed daily routine.

This has a direct commercial implication. The category may currently be positioned in consumers’ minds as an occasional remedy rather than an everyday product. Factors such as premium pricing or limited availability may also play a role [70]. Brands seeking regular consumption would need to establish these beverages as accessible, everyday choices, although whether this is achievable, or whether situational use is the category’s natural pattern, requires further research using behavioural data.

### 4.7. Socio-Cultural Context and Stress Prevalence

National stress levels offer one real-world explanation for the differences between markets. Approximately 33% of New Zealand adults report daily stress [71], compared with more than 50% in India [69] and 60% in Malaysia [70]. Where stress is more prevalent, stress-reducing beverages may shift from a lifestyle option to a functional need. Consistent with this, Indian participants prioritised proven efficacy, and Malaysian participants sought trustworthy, situation-specific solutions. New Zealand participants, with lower baseline stress, responded instead to convenience and promotions, treating the beverages as an optional purchase. As stress prevalence was not measured at the individual level in this study, this explanation should be regarded as contextual rather than tested.

### 4.8. Cross-Cultural Variability in Intentions to Purchase Stress-Reducing Beverages

Taken together, the findings show why combining the two frameworks is useful in practice. The TPB constructs explain what is common across markets: in every country, young consumers need to believe the product works and to feel that people close to them approve. The TCV constructs explain what differs: the specific product-level reasons that turn a positive view into an intention to buy. Without the value-based constructs, the model would miss the most commercially relevant differences, namely efficacy in India, trust and situational fit in Malaysia, and convenience and price in New Zealand. By integrating both psychosocial and value-driven determinants, this research extends the work of Natarajan et al. [31] and provides a practical account of the emerging stress-reducing beverage segment. Because demographic covariates largely did not significantly alter purchase intention (with the exceptions of ethnicity in the Indian subsample and educational qualification in the Malaysian subsample), the demographic profile of each cohort is best understood as describing who holds each country’s driver pattern, rather than as its cause. Read this way, the three cohorts translate into distinct market personas:

The Health-Pragmatic Buyer (India). This cohort is comparatively older than the other two samples, more often married, and the most highly educated, and is familiar with Ayurvedic ingredients such as Tulsi and Ashwagandha. Their purchase intention is anchored in functional value, so they respond to evidence of efficacy and safety cues such as additive-free labelling. The significant effect of ethnicity within the Indian subsample further suggests that messaging should be tailored to different regional and ethnic groups, such as Dravidian and Indo-Aryan consumers, rather than treating India as a single market.

The Trust-Seeking Young Woman (Malaysia). This cohort is young, predominantly female (73.18%) mostly single, often living with parents, and degree-educated. Their intentions depend on initial trust and conditional value rather than on health claims alone. For this persona, authenticity signals and low-risk trial opportunities (Section 4.3) are prerequisites for adoption.

The Price-Conscious Convenience Seeker (New Zealand). This cohort is young, with comparatively lower reported income and educational attainment. Their intentions are driven by perceived behavioural control, conditional value, and, notably, subjective norms. For this persona, accessible pricing, promotions, and wide retail availability matter more than health or trust cues, and peer-oriented messaging may reinforce purchase decisions.

These personas give regional marketers an actionable starting point for segment-specific positioning, which Section 4.9 builds on.

### 4.9. Practical Implications and Industry Recommendations

The findings of this study provide a strategic framework for the development and marketing of stress-reducing beverages across the Asia-Pacific region, emphasising the need for culturally tailored strategies that reflect the value-based and normative determinants identified across the three markets. Because attitude was a significant driver of purchase intention in all three countries, marketers of stress-reducing beverages should prioritise educational campaigns that clearly communicate the physiological and psychological benefits of their products. Brands should avoid generic marketing and instead highlight specific stress-relief mechanisms. Furthermore, promotional strategies must be culturally tailored rather than standardised. For example, price-based promotions were a significant driver in Malaysia and New Zealand but not in India, where participants responded neutrally to discounts. In the Indian market, campaigns should therefore lead with evidence of efficacy instead. Social value was non-significant in all three countries, so status-oriented or influencer-led positioning is unlikely to be effective in any of these markets. The recommendations below are organised around the three consumer personas identified in Section 4.8. The Health-Pragmatic Buyer (India). For the Indian market, success depends on highlighting functional efficacy. Because Indian consumers prioritise tangible health utility, manufacturers should emphasise transparent ingredient sourcing and lead with evidence-based health claims [21] Manufacturers can also leverage high cultural familiarity with traditional ingredients such as Tulsi and Ashwagandha to reinforce this functional value, consistent with evidence that health consciousness drives the purchase of Ayurvedic products in India [49], rather than relying on trust-building mechanisms, which were non-significant for Indian participants. As this was the most highly educated of the three cohorts, detailed, evidence-based product information is likely to be well received.

The Trust-Seeking Young Woman (Malaysia). In Malaysia, market penetration requires establishing the product as a reliable, situation-specific solution. Since initial trust is a significant driver in this market, establishing brand authenticity through transparent health labels and independent certifications is essential for adoption [48]. Certification is an established first signal of confidence for Malaysian consumers [50]. However, the cohort was ethnically diverse (55.00% Chinese, 31.36% Malay), so halal certification should be complemented by regulatory or independent quality marks that carry weight across ethnic groups. Because conditional value also dictates purchase decisions, marketing strategies must emphasise contextual relevance. Positioning these beverages as a functional alternative to caffeinated energy drinks during periods of study and early-career pressure, consistent with participants’ reported willingness to switch from conventional energy drinks (Section 3.3), combined with strategic sampling on university campuses and in high-stress urban locations, will likely be more effective than broad, non-specific advertising. Low-commitment formats, such as single-serve packs and introductory discounts, allow this persona to verify a product before committing to repeat purchase. The Price-Conscious Convenience Seeker (New Zealand). For the New Zealand market, strategies must align with conditional value and perceived behavioural control. Responsiveness to discounts and promotional offers is particularly high among New Zealanders, suggesting that price-based incentives are critical for initial market penetration [19]. This fits the New Zealand retail environment, where nearly half of household grocery purchases are made on promotion but healthier products are less likely to be promoted [51], leaving room for functional beverages to compete on price. Marketing campaigns should encourage switching behaviour, positioning stress-reducing beverages as a convenient substitute for conventional energy drinks during periods of environmental stress [29,30]. Given that New Zealand consumers prioritise personal control and ease of access, ensuring high product visibility in mainstream supermarkets and convenience retail is vital to transforming occasional intention into more regular purchase. Subjective norms were also significant in New Zealand, so peer-oriented tactics such as referral offers or shared-purchase promotions may reinforce price and convenience messages.

### 4.10. Limitations and Future Research

While this study provides insights, there are several limitations. First, the sample mainly comprised young adults aged 18 to 25, which may limit the generalisability of these findings to older demographic groups. Second, the survey instrument did not explicitly measure urban versus rural place of living. Given the online, English language questionnaire administered, the findings are likely skewed toward urban, educated populations and may not fully represent rural consumer intentions. Furthermore, the survey relied on category definitions rather than localized brand examples, which may have impacted how respondents conceptualized the product. Additionally, the three cohorts from different countries differed demographically, where the Indian participants skewed older and more often lived without parents, the Malaysian participants were predominantly female and single, and the New Zealand participants reported lower educational attainment. Since these factors covary with country, the cross-cultural differences observed cannot be fully separated from these underlying demographic differences.

Methodologically, all survey items were worded in a positive direction to prevent respondent confusion and preserve scale reliability across diverse linguistic groups; however, we acknowledge that this design introduces the potential for acquiescence bias. More generally, because all data were collected through cross-sectional self-report questionnaires, the findings may be affected by common-method bias. However, Harman’s single-factor test showed that no single factor accounted for the majority of the variance in any of the three samples (36.72–44.57%), suggesting that common-method bias was not a pervasive issue in this dataset. Furthermore, the survey instrument solely measured general purchase intent and planned consumption frequency rather than actual subsequent behaviour; therefore this study cannot confirm whether these intentions translate into action. The findings therefore remain subject to the well-documented intention-behaviour gap, whereby stated dietary intentions do not consistently predict actual consumer choices.

To address these limitations, future research should use multilingual surveys and quota sampling to capture a more representative, demographically balanced cross-cultural profile. Collecting predictor and outcome measures at different time points or from different sources would further reduce the risk of common-method bias. Finally, longitudinal research incorporating actual retail sales metrics, such as supermarket checkout scanner data, or consumption diaries is necessary to validate these behavioural drivers, move beyond self-reported intentions, and confirm long-term habit formation.

## 5. Conclusions

While attitudes and perceived behavioural control predicted the intention to purchase stress-reducing beverages in New Zealand and India, the values driving these decisions differed among the three national cohorts. Functional value was the main motivator in India, where consumers prioritise health efficacy and obvious results. In contrast, purchase intention in the Malaysian cohort was driven by conditional value and initial trust, highlighting a clear demand for situational relevance and brand authenticity. New Zealanders showed a distinct sensitivity to conditional value, with promotional incentives often carrying more weight than intrinsic health benefits. The non-significant influence of social and epistemic values suggests that consumers treat these products as personal health interventions rather than status symbols. However, because the three national samples differed in age, gender, education and income, and were recruited through non-probability sampling, these country-level differences should be interpreted as differences between the sampled cohorts rather than as established cultural effects. With this caveat, the findings suggest that the industry should consider moving from standardised global marketing toward market-specific strategies that address the specific functional, trust-based, and situational requirements of consumers in different Asia-Pacific countries.

## Figures and Tables

**Table 1:** Measurement items for the Theory of Planned Behaviour and Theory of Consumption Values constructs to determine purchase behaviour of stress-reducing beverages.

Constructs (TPB and TCV)	Statements	Scale
Attitudes (ATT)	I think drinking stress-reducing drinks in the future is enjoyable (ATT1) I think drinking stress-reducing drinks will be a pleasant sensory experience (ATT2) I think it is important to drink stress-reducing drinks in the future (ATT3) It is beneficial to drink stress-reducing drinks in the future to improve my health (ATT4) I think consuming stress-reducing drinks will help me to face stressful situations (ATT5)	1 = Extremely unlikely to 5 = Extremely likely
Subjective Norms (SN)	My friends and family agree I should consume stress-reducing drinks (SN1) My family and friends think and feel positively when I consume stress-reducing drinks (SN2) My friends and family will buy and consume stress-reducing drinks (SN3)	1 = Strongly disagree to 5 = Strongly agree 1 = Very negative to 5 = Very positive 1 = Extremely unlikely to 5 = Extremely likely
Perceived Behavioural Control Items (PBC)	I will drink stress-reducing drinks completely by myself without any external influences from family and friends (PBC1) I will decide entirely for myself to drink stress-reducing drinks (PBC2) I feel empowered when I buy stress-reducing drinks (PBC3) The decision to buy stress-reducing drinks will be completely up to myself (PBC4)	1 = Extremely unlikely to 5 = Extremely likely
Behavioural Intention Items (BI)	I will for sure drink stress-reducing drinks in the future (BI1) How often do you plan to drink stress-reducing drinks? (BI2) I would also recommend others to buy and consume stress-reducing drinks (BI3)	1 = Strongly disagree to 5 = Strongly agree 1 = 0–1 day/week, 2 = 2–3 days/week, 3 = 4–5 days/week, 4 = 6–7 days/week 1 = Strongly disagree to 5 = Strongly agree
Functional Value (FV)	Stress-reducing drinks improve health and reduce stress (FV1) Stress-reducing drinks are healthier than energy drinks (FV2) Stress-reducing drinks are free from food additives (FV3) Stress-reducing drinks are strong in product safety (FV4)	1 = Strongly disagree to 5 = Strongly agree
Social Value (SV)	When I consume stress-reducing drinks I feel admired by other people (SV1) I feel accepted by others when I buy stress-reducing drinks (SV2) I feel respected by others when buying stress-reducing drinks (SV3) Consuming stress-reducing drinks supports me in how people see and evaluate me (SV4)	1 = Strongly disagree to 5 = Strongly agree
Conditional Value (CV)	I decide to purchase stress-reducing drinks when they are offered with discounts or with promotional offers (CV1) I consume stress-reducing drinks instead of conventional drinks like energy drinks under aggravating environmental circumstances (CV2) I prefer consuming stress-reducing drinks instead of conventional drinks like energy drinks under subsidiary conditions (CV3)	1 = Strongly disagree to 5 = Strongly agree
Epistemic Value (EV)	I search for information about stress-reducing drinks before buying them (EV1) I prefer to gain information about stress-reducing drinks and compare all available options of stress-reducing drinks before I decide to acquire them (EV2) Consuming stress-reducing drinks will be a new adventure for me (EV3)	1 = Strongly disagree to 5 = Strongly agree
Initial Trust (IT)	I believe that stress-reducing drinks work to reduce stress (IT1) I believe stress-reducing drinks are trustworthy (IT2) I believe stress-reducing drinks are authentic (IT3)	1 = Strongly disagree to 5 = Strongly agree

**Table 2:** Sociodemographic and general information of participants from New Zealand, India, and Malaysia.

New Zealand				India				Malaysia			
Variable	Group	Number	Percentage	Variable	Group	Number	Percentage	Variable	Group	Number	Percentage
Age	18–25	141	64.09	Age	18–25	112	50.91	Age	18–25	147	66.82
	26–35	42	19.09		26–35	69	31.36		26–35	28	12.73
	36–45	20	9.09		36–45	24	10.91		36–45	26	11.82
	46–55	6	2.73		46–55	12	5.45		46–55	13	5.91
	56–65	9	4.09		56–65	2	0.91		56–65	6	2.73
	Prefer not to say	2	0.91		Prefer not to say	1	0.45		Prefer not to say	0	0.00
Gender	Male	115	52.27	Gender	Male	97	44.09	Gender	Male	55	25.00
	Female	101	45.91		Female	119	54.09		Female	161	73.18
	Non-binary/third gender	3	1.36		Non-binary/third gender	1	0.45		Non-binary/third gender	0	0.00
	Prefer not to say	1	0.45		Prefer not to say	3	1.36		Prefer not to say	4	1.82
Ethnicity	European	79	35.91	Ethnicity	Indo-Aryans	59	26.82	Ethnicity	Malay	69	31.36
	Maori	7	3.18		Dravidians	143	65.00		Chinese	121	55.00
	Pacific Peoples	14	6.36		Mongoloid populations	6	2.73		Indian	15	6.82
	Asian	77	35.00		Other Ethnicity	12	5.45		Indigenous/Orang Asli	3	1.36
	Middle Eastern/Latin American/African	23	10.45		-	-	-		Eurasian	1	0.45
	Other Ethnicity	20	9.09		-	-	-		Other Ethnicity	11	5.00
	Residual Categories	0	0		-	-	-		-	-	-
Marital status	Single	126	57.27	Marital status	Single	118	53.64	Marital status	Single	167	75.91
	Non-marital partnership	50	22.73		Non-marital partnership	14	6.36		Non-marital partnership	9	4.09
	Married	32	14.55		Married	71	32.27		Married	37	16.82
	Separate/divorced	5	2.27		Separate/divorced	5	2.27		Separate/divorced	3	1.36
	Widower	2	0.91		Widower	6	2.73		Widower	0	0.00
	Prefer not to say	5	2.27		Prefer not to say	6	2.73		Prefer not to say	4	1.82
Household composition I	I live alone	26	11.82	Household composition I	I live alone	45	20.45	Household composition I	I live alone	22	10.00
	Spouse/partner	36	16.36		Spouse/partner	37	16.82		Spouse/partner	28	12.73
	Child(ren) aged under 18	9	4.09		Child(ren) aged under 18	21	9.55		Child(ren) aged under 18	11	5.00
	Child(ren) aged over 18	3	1.36		Child(ren) aged over 18	14	6.36		Child(ren) aged over 18	11	5.00
	Parents	96	43.64		Parents	55	25.00		Parents	113	51.36
	Flatmate/s	39	17.73		Flatmate/s	34	15.45		Flatmate/s	15	6.82
	Other	7	3.18		Other	8	3.64		Other	12	5.45
	Prefer not to say	4	1.82		Prefer not to say	6	2.73		Prefer not to say	8	3.64
Household composition II	1–2 people	61	27.73	Household composition II	1–2 people	78	35.45	Household composition II	1–2 people	57	25.91
	3–4 people	100	45.45		3–4 people	96	43.64		3–4 people	111	50.45
	more than 5 people	55	25.00		more than 5 people	39	17.73		more than 5 people	46	20.91
	Prefer not to say	4	1.82		Prefer not to say	7	3.18		Prefer not to say	6	2.73
Academic qualification	High school or less	33	15.00	Academic qualification	High school or less	3	1.36	Academic qualification	High school or less	12	5.45
	High school diploma	81	36.82		High school diploma	25	11.36		High school diploma	36	16.36
	Bachelor degree	61	27.73		Bachelor degree	102	46.36		Bachelor degree	138	62.73
	Master degree	29	13.18		Master degree	75	34.09		Master degree	13	5.91
	Doctorate degree (PhD)	9	4.09		Doctorate degree (PhD)	11	5.00		Doctorate degree (PhD)	20	9.09
	Prefer not to say	7	3.18		Prefer not to say	4	1.82		Prefer not to say	1	0.45
Yearly income ^b^	Less than NZ$29,999	121	55.00	Yearly income-	Less than 50,000 rs	36	16.36	Yearly income	Less than RM 29,999	88	40.00
	NZ$30,000–NZ$49,999	23	10.45		50,000 rs—3 L rs	28	12.73		RM 30,000–RM 49,999	15	6.82
	NZ$50,000–NZ$79,999	12	5.45		300,001—5 L	47	21.36		RM 50,000–RM 79,999	28	12.73
	NZ$80,000–NZ$99,999	9	4.09		500,001—10 L	62	28.18		RM 80,000–RM 99,999	8	3.64
	NZ$100,000 or more	18	8.18		more than 10 L	6	2.73		RM 100,000–149,999	5	2.27
	Prefer not to say	37	16.82		Prefer not to say	41	18.64		RM 150,000 or more	7	3.18
	-	-	-		-	-	-		Prefer not to say	69	31.36
Beverages consumption habits ^a^	Shot	51	11.51	Beverages consumption habits ^a^	Shot	5	2.27	Beverages consumption habits ^a^	Shot	32	7.98
	Juice	148	33.41		Juice	92	41.82		Juice	171	42.64
	Energy drink	100	22.57		Energy drink	42	19.09		Energy drink	49	12.22
	Fermented beverages like kombucha	44	9.93		Fermented beverages like kombucha	2	0.91		Fermented beverages like kombucha	61	15.21
	Vinegar	19	4.29		Vinegar	0	0.00		Vinegar	16	3.99
	Others	81	18.28		Others	79	35.91		Others	72	17.96
Supplements Familiarity ^a^	Ashwagandha	81	6.65	Supplements Familiarity ^a^	Ashwagandha	116	9.20	Supplements Familiarity ^a^	Ashwagandha	36	3.25
	Ginseng	118	9.69		Ginseng	54	4.28		Ginseng	148	13.38
	Lavender	110	9.03		Lavender	128	10.15		Lavender	80	7.23
	Jujube/red dates	44	3.61		Jujube/red dates	76	6.03		Jujube/red dates	115	10.40
	Holy basil/tulsi	44	3.61		Holy basil/tulsi	145	11.50		Holy basil/tulsi	35	3.16
	Schisandra	7	0.57		Schisandra	10	0.79		Schisandra	4	0.36
	Licorice	80	6.57		Licorice	12	0.95		Licorice	32	2.89
	Turmeric	154	12.64		Turmeric	172	13.64		Turmeric	129	11.66
	Reishi mushroom	19	1.56		Reishi mushroom	189	14.99		Reishi mushroom	21	1.90
	Garlic	166	13.63		Garlic	33	2.62		Garlic	151	13.65
	Ginger	180	14.78		Ginger	184	14.59		Ginger	170	15.37
	Fish oil	162	13.30		Fish oil	118	9.36		Fish oil	159	14.38
	Others	53	4.35		Others	24	1.90		Others	26	2.35
Supplements intake I	Daily	60	27.27	Supplements intake I	Daily	38	17.27	Supplements intake I	Daily	57	25.91
	Weekly	30	13.64		Weekly	60	27.27		Weekly	35	15.91
	Monthly	13	5.91		Monthly	36	16.36		Monthly	17	7.73
	Rarely	79	35.91		Rarely	49	22.27		Rarely	97	44.09
	Never	38	17.27		Never	37	16.82		Never	14	6.36
Supplements intake II ^a^	Capsules	100	23.92	Supplements intake II ^a^	Capsules	86	39.09	Supplements intake II ^a^	Capsules	127	27.37
	Tablets	80	19.14		Tablets	39	17.73		Tablets	89	19.18
	Powder	49	11.72		Powder	12	5.45		Powder	47	10.13
	Tinctures	0	0.00		Tinctures	0	0.00		Tinctures	1	0.22
	Drinks	83	19.86		Drinks	21	9.55		Drinks	97	20.91
	Food	75	17.94		Food	54	24.55		Food	95	20.47
	Others	31	7.42		Others	8	3.64		Others	8	1.72

^a^ Multiple answers were possible for research participants. ^b^ Exchange rates as of 24 July 2026 (mid-market rates, Xe.com): 1 NZD ≈ ₹55.79 INR ≈ RM2.37. “L” denotes lakh (1 lakh = 100,000 INR) [64].

**Table 3:** Distribution scores of responses from participants in New Zealand, India, and Malaysia for the constructs of the Theory of Planned Behaviour and the constructs of the Theory of Consumption Values to investigate the purchase behaviour of stress-reducing drinks in New Zealand, India, and Malaysia.

		1–2 Score Distribution (%)			3 Score Distribution (%)			4–5 Score Distribution (%)			Scale
Constructs	Question	New Zealand	India	Malaysia	New Zealand	India	Malaysia	New Zealand	India	Malaysia	
Attitudes	ATT1	17.27	19.55	13.64	25.91	21.82	21.36	56.82	58.64	65.00	1 = Extremely unlikely to 5 = Extremely likely
	ATT2	14.55	12.73	12.27	24.09	25.00	20.45	61.36	62.27	67.27	
	ATT3	25.00	15.45	14.55	28.64	23.18	31.82	46.36	61.36	53.64	
	ATT4	19.09	19.09	13.64	31.82	16.82	27.27	49.09	64.09	59.09	
	ATT5	22.73	18.64	14.55	27.73	25.45	26.36	49.55	55.91	59.09	
Subjective norms	SN1	29.55	35.91	19.55	44.55	42.73	50.91	25.91	21.36	29.55	1 = Strongly disagree to 5 = Strongly agree
	SN2	16.82	34.09	12.27	59.55	45.00	54.09	23.64	20.91	33.64	1 = Very negative to 5 = Very positive
	SN3	26.36	35.91	24.55	35.91	42.73	34.55	37.73	21.36	40.91	1 = Extremely unlikely to 5 = Extremely likely
Perceived Behavioural Control Items	PBC1	24.09	17.27	17.27	27.73	27.27	25.00	48.18	55.45	57.73	1 = Strongly agree to 5 = Strongly disagree
	PBC2	15.91	12.73	8.18	20	22.73	23.64	64.09	64.55	68.18	
	PBC3	25.91	20.91	15.45	45.91	41.36	46.82	28.18	37.73	37.73	
	PBC4	11.36	10.45	5.00	20.00	22.27	24.09	68.64	67.27	70.91	
Behavioural Intention Items	BI1	25.91	19.09	13.64	37.27	40.00	30.45	36.82	40.91	55.91	1 = Extremely unlikely to 5 = Extremely likely
	BI2	90.00 ^b^	91.82 ^b^	90.45 ^b^	6.36 ^b^	5.45 ^b^	5.91 ^b^	3.64 ^b^	2.73 ^b^	3.64 ^b^	1 = 0–1 day/week, 2 = 2–3 days/week, 3 = 4–5 days/week, 4 = 6–7 days/week
	BI3	21.82	19.55	12.73	41.82	44.09	37.27	36.36	36.36	50.00	1 = Extremely unlikely to 5 = Extremely likely
Functional Value	FV1	17.73	15.45	6.36	36.82	33.64	32.73	45.45	50.91	60.91	1 = Strongly disagree to 5 = Strongly agree
	FV2	6.82	12.27	8.18	29.09	28.64	42.73	64.09	59.09	49.09	
	FV3	17.73	10.00	24.55	53.18	38.64	37.73	29.09	51.36	37.73	
	FV4	11.36	10.91	13.64	55.91	41.82	49.55	32.73	47.27	36.82	
Social Value	SV1	34.55	36.82	37.27	49.55	38.64	43.64	15.91	24.55	19.09	1 = Strongly disagree to 5 = Strongly agree
	SV2	28.64	40.00	34.55	51.82	34.55	45.00	19.55	25.45	20.45	
	SV3	30.00	37.27	34.55	50.91	37.27	46.82	19.09	25.45	18.64	
	SV4	32.27	35.91	35.91	48.64	39.55	43.64	19.09	24.55	20.45	
Conditional Value	CV1	19.55	15.45	8.18	28.18	58.64	24.55	52.27	25.91	67.27	1 = Strongly disagree to 5 = Strongly agree
	CV2	27.27	20.00	16.82	34.55	54.09	38.18	38.18	25.91	45.00	
	CV3	21.36	12.73	12.27	39.55	60.91	40.00	39.09	26.36	47.73	
Epistemic Value	EV1	27.27	15.91	9.55	30.00	22.73	20.45	42.73	61.36	70.00	1 = Strongly disagree to 5 = Strongly agree
	EV2	20.91	10.00	8.64	29.55	20.91	16.82	49.55	69.09	74.55	
	EV3	16.82	10.91	7.73	27.73	26.36	26.82	55.45	62.73	65.45	
Initial Trust	IT1	13.18	14.55	10.91	40.91	28.18	24.55	45.91	57.27	64.55	1 = Strongly disagree to 5 = Strongly agree
	IT2	16.82	11.82	15.91	46.36	34.09	41.82	36.82	54.09	42.27	
	IT3	15.45	11.36	16.82	48.64	39.09	37.73	35.91	49.55	45.45	

^b^ The scale used for BI2 is a scale from 1 to 4 with 1 = 0–1 day/week, 2 = 2–3 days/week, 3 = 4–5 days/week, 4 = 6–7 days/week. 1–2 score distribution includes 0–1 day/week and 2–3 days/week. 3 score distribution consists of 4–5 days/week and 4–5 score distribution presents 6–7 days/week.

**Table 4:** Results of constructs showing path coefficients (β), standard errors, t-values, *p*-values, and effect sizes (f^2^) for the structural models of New Zealand, India, and Malaysia.

Constructs	Path Coefficient (β)	Standard Error	t-Value	*p*-Value	Effect Size (f^2^)	Lower Bound	Upper Bound	Inner Model VIF
SN → PBC								
NZ	0.565	0.056	10.101	0.000	0.468	0.462	0.678	1.000
India	0.406	0.062	6.562	0.000	0.198	0.327	0.513	1.000
Malaysia	0.319	0.062	5.139	0.000	0.122	0.126	0.476	1.000
SN → ATT								
NZ	0.586	0.055	10.669	0.000	0.522	0.465	0.668	1.000
India	0.410	0.062	6.637	0.000	0.202	0.292	0.535	1.000
Malaysia	0.426	0.062	6.865	0.000	0.217	0.284	0.603	1.000
SN → BI								
NZ	0.239	0.059	4.083	0.000	0.079	0.107	0.337	1.991
India	0.192	0.048	4.006	0.000	0.076	0.096	0.308	1.633
Malaysia	0.146	0.059	2.455	0.015	0.029	0.051	0.301	1.930
PBC → BI								
NZ	0.216	0.061	3.529	0.001	0.059	0.075	0.379	2.161
India	0.159	0.057	2.809	0.005	0.037	0.031	0.310	2.292
Malaysia	−0.004	0.059	−0.063	0.950	0.000	−0.125	0.159	1.910
ATT → BI								
NZ	0.170	0.061	2.812	0.005	0.037	0.042	0.272	2.126
India	0.261	0.055	4.730	0.000	0.106	0.143	0.402	2.175
Malaysia	0.345	0.061	5.686	0.000	0.153	0.089	0.573	2.023
FV → BI								
NZ	0.089	0.063	1.396	0.164	0.009	−0.097	0.259	2.333
India	0.338	0.063	5.389	0.000	0.138	0.231	0.506	2.809
Malaysia	0.095	0.071	1.326	0.186	0.008	−0.081	0.276	2.796
IT → BI								
NZ	0.051	0.061	0.838	0.403	0.003	−0.101	0.227	2.145
India	0.051	0.081	0.629	0.530	0.002	−0.176	0.187	4.684
Malaysia	0.193	0.078	2.468	0.014	0.029	0.029	0.398	3.371
SV → BI								
NZ	0.012	0.054	0.228	0.820	0.000	−0.109	0.124	1.689
India	0.080	0.047	1.704	0.090	0.014	−0.022	0.179	1.568
Malaysia	0.061	0.050	1.217	0.225	0.007	−0.048	0.202	1.390
CV → BI								
NZ	0.186	0.063	2.950	0.004	0.041	0.034	0.360	2.304
India	0.040	0.054	0.746	0.456	0.003	−0.077	0.153	2.063
Malaysia	0.209	0.060	3.497	0.001	0.058	0.099	0.343	1.959
EV → BI								
NZ	0.055	0.051	1.079	0.282	0.006	−0.035	0.163	1.525
India	−0.078	0.057	−1.368	0.173	0.009	−0.196	0.068	2.319
Malaysia	−0.049	0.055	−0.891	0.374	0.004	−0.167	0.055	1.636

Q^2^ values were calculated for endogenous constructs: ATT1: 0.116, ATT2: 0.129, ATT3: 0.183, ATT4: 0.208, ATT5: 0.226, PBC1: 0.137, PBC2: 0.248, PBC3: 0.098, PBC4: 0.048, BI1: 0.449, BI2: 0.120, BI3: 0.465. All Q^2^ values > 0, indicating that the model predicts new data better than a simple mean-based guess and thus has predictive relevance.

**Table 5:** Measurement Model Assessment: Dillon-Goldstein’s rho, Cronbach’s alpha, AVE, GoF, and GoF (Bootstrapp) for TPB and TCV Constructs Across New Zealand, India, and Malaysia.

	Dillon-Goldstein’s Rho			Cronbach’s Alpha			AVE			GoF			GoF (Bootstrapp)			Composite Reliability (rho_a)			Composite Reliability (rho_c)		
TPB and TCV Constructs	NZ	India	Mal	NZ	India	Mal	NZ	India	Mal	NZ	India	Mal	NZ	India	Mal	NZ	India	Mal	NZ	India	Mal
Attitudes	0.903	0.946	0.918	0.865	0.929	0.888	0.649	0.777	0.689	-	-	-	-	-	-	0.872	0.937	0.898	0.902	0.946	0.917
Subjective Norms	0.899	0.923	0.889	0.831	0.875	0.813	0.748	0.799	0.728	-	-	-	-	-	-	0.832	0.885	0.815	0.899	0.923	0.889
Perceived Behavioural Control Items	0.869	0.882	0.882	0.796	0.819	0.820	0.618	0.636	0.633	-	-	-	-	-	-	0.836	0.861	0.888	0.864	0.875	0.872
Behavioural Intention Items	0.864	0.818	0.830	0.762	0.662	0.687	0.680	0.605	0.628	-	-	-	-	-	-	0.814	0.783	0.807	0.862	0.813	0.827
Functional Value	0.874	0.936	0.871	0.808	0.909	0.801	0.632	0.785	0.621	-	-	-	-	-	-	0.825	0.917	0.826	0.873	0.936	0.868
Social Value	0.949	0.956	0.958	0.928	0.939	0.941	0.824	0.845	0.849	-	-	-	-	-	-	0.931	0.943	0.949	0.949	0.956	0.957
Conditional Value	0.887	0.916	0.853	0.808	0.861	0.740	0.724	0.783	0.658	-	-	-	-	-	-	0.813	0.861	0.744	0.887	0.916	0.852
Epistemic Value	0.814	0.898	0.891	0.652	0.829	0.814	0.603	0.741	0.731	-	-	-	-	-	-	0.711	0.856	0.822	0.814	0.895	0.890
Initial Trust	0.905	0.946	0.905	0.841	0.914	0.842	0.760	0.854	0.759	-	-	-	-	-	-	0.853	0.916	0.854	0.905	0.946	0.904
-	-	-	-	-	-	-	-	-	-	0.546	0.512	0.607	0.546	0.518	0.618						

SRMR: NZ: Saturated Model: 0.069, Estimated Model: 0.106, India: Saturated Model: 0.072 Estimated Model: 0.194 Malaysia: Saturated Model: 0.091 Estimated Model: 0.11.

**Table 6:** Results of multigroup *t*-test analysis comparing the purchase behaviour of stress-reducing beverages across TPB and TCV variables among participants from New Zealand, India, and Malaysia.

Path Coefficients with *p*-Values Obtained from Multigroup *t* Test Analysis Comparing the Impact of Latent Variable Between Two DIFFERENT Countries.			
**Latent Variables**	New Zealand vs. India	New Zealand vs. Malaysia	India vs. Malaysia
**Subjective norms → perceived behavioural control**	0.159 *	0.246 *	**0.087**
**Subjective norms → attitudes**	**0.047**	0.093	**0.046**
**Subjective norms → behavioural intention**	0.047	0.093	0.046
**Perceived behavioural control → behavioural intention**	0.057	0.220 *	0.163 *
**Attitudes → behavioural intention**	−0.091	−0.175	−0.084
**Functional value → behavioural intention**	−0.249 **	−0.006	0.243 *
**Initial trust → behavioural intention**	0.000	−0.142	−0.142
**Social value → behavioural intention**	−0.068	−0.049	0.019
**Conditional value → behavioural intention**	0.146	−0.023	−0.169 *
**Epistemic value → behavioural intention**	0.133	0.104	−0.029

Significant path coefficients have been bolded. * indicates a 5% significance and ** indicates a 1% significance.

## Data Availability

The original contributions presented in this study are included in the article/supplementary material. Further inquiries can be directed to the corresponding author.

## Supplementary Materials

The following supporting information can be downloaded at: https://www.mdpi.com/article/10.3390/foods15213781/s1, Table S1: Weight contribution of manifest variables to latent variables. Table S2. Distribution Scores for Lifestyle and Wellness Practices. Table S3. Outer Loadings Results. Table S4. Results of Discriminant Validity. Table S5. Detailed Measurement Invariance Assessment (MICOM) Results by Construct and Country Pair. Table S6. Discriminant validity assessed using the heterotrait–monotrait ratio (HTMT) for the TPB and TCV constructs across New Zealand, India and Malaysia. Table S7. Covariate-adjusted sensitivity analysis: effects of demographic variables on behavioural intention in the New Zealand, Indian and Malaysian samples. Table S8. Sensitivity analysis of the behavioural intention (BI) composite: structural path estimates with and without item BI2.
