# Supplementary material for: Integrating the Theory of Planned Behaviour and Theory of Consumption Values to Explain Purchase Intentions for Stress-Reducing Beverages: A Cross-Cultural Study of New Zealand, India, and Malaysia
Source: Foods. 2026 Oct 22;15(21):3781. doi: 10.3390/foods15213781 (PMC13762870; doi:10.3390/foods15213781)
Supplement: Supplementary file 1 [file foods-15-03781-s001.zip › foods-4569589-supplementary.pdf]

Table S1. Weight contribution of manifest variables to latent variables

Latent variables	Manifest variable	Weight		
		New Zealand	India	Malaysia
Attitudes (ATT)	I think drinking stress-reducing drinks in the future is enjoyable (ATT1)	0.221	0.193	0.196
	I think drinking stress-reducing drinks will be a pleasant sensory experience (ATT2)	0.233	0.200	0.201
	I think it is important to drink stress-reducing drinks in the future (ATT3)	0.272	0.225	0.277
	It is beneficial to drink stress-reducing drinks in the future to improve my health (ATT4)	0.282	0.241	0.255
	I think consuming stress-reducing drinks will help me to face stressful situations (ATT5)	0.230	0.273	0.274
Subjective Norms (SN)	My friends and family agree I should consume stress-reducing drinks (SN1)	0.390	0.330	0.402
	My family and friends think and feel positively when I consume stress-reducing drinks (SN2)	0.384	0.371	0.368
	My friends and family will buy and consume stress-reducing drinks (SN3)	0.382	0.418	0.403
Perceived Behavioural Control Items (PBC)	I will drink stress-reducing drinks completely by myself without any external influences from family and friends (PBC1)	0.374	0.288	0.335
	I will decide entirely for myself to drink stress-reducing drinks (PBC2)	0.288	0.266	0.271
	I feel empowered when I buy stress-reducing drinks (PBC3)	0.398	0.457	0.465
	The decision to buy stress-reducing drinks will be completely up to myself (PBC4)	0.191	0.243	0.163
Behavioural Intention Items (BI)	I will for sure drink stress-reducing drinks in the future (BI1)	0.285	0.541	0.490
	How often do you plan to drink stress-reducing drinks?(BI2)	0.444	0.202	0.203
	I would also recommend others to buy and consume stress-reducing drinks (BI3)	0.462	0.472	0.502
Functional Value (FV)	Stress-reducing drinks improve health and reduce stress (FV1)	0.397	0.325	0.421
	Stress-reducing drinks are healthier than energy drinks (FV2)	0.269	0.263	0.293
	Stress-reducing drinks are free from food additives (FV3)	0.303	0.284	0.275
	Stress-reducing drinks are strong in product safety (FV4)	0.287	0.257	0.276
Social Value (SV)	When I consume stress-reducing drinks I feel admired by other people (SV1)	0.258	0.299	0.246
	I feel accepted by others when I buy stress-reducing drinks (SV2)	0.290	0.265	0.312
	I feel respected by others when buying stress-reducing drinks (SV3)	0.284	0.245	0.254
	Consuming stress-reducing drinks support me in how people see and assess me (SV4)	0.268	0.280	0.273
Conditional Value (CV)	I decide to purchase stress-reducing drinks when they are offered with discounts or with promotional offers (CV1)	0.364	0.377	0.415
	I consume stress-reducing drinks instead of conventional drinks like energy drinks under aggravating environmental circumstances (CV2)	0.411	0.376	0.446
	I prefer consuming stress-reducing drinks instead of conventional drinks like energy drinks under subsidiary conditions (CV3)	0.399	0.377	0.372
Epistemic Value (EV)	I search for information about stress-reducing drinks before buying them (EV1)	0.468	0.329	0.336
	I prefer to gain information about stress-reducing drinks and compare all available options of stress-reducing drinks before I decide to acquire them (EV2)	0.487	0.357	0.431
	Consuming stress-reducing drinks will be a new adventure for me (EV3)	0.312	0.475	0.405

Initial Trust (IT)	I believe that stress-reducing drinks work to reduce stress (IT1)<br>I believe stress-reducing drinks are trustworthy (IT2)<br>I believe stress-reducing drinks are authentic (IT3)	0.395<br>0.412<br>0.336	0.351<br>0.374<br>0.357	0.413<br>0.413<br>0.320

Table S2. Distribution Scores for Lifestyle and Wellness Practices

		1-2 score distribution (%)			3 score distribution (%)			4-5 score distribution (%)			Scale
**Constructs**	Question	New Zealand	India	Malaysia	New Zealand	India	Malaysia	New Zealand	India	Malaysia	
**General information (exercising and mindful practices)[a]**	Exercises	11.36	21.36	26.82	33.64	30.91	47.27	55.00	47.73	25.91	1= never to 5= always
	Meditation	70.91	57.27	76.36	15.91	23.18	15.00	13.18	19.55	8.64	
	Psychotherapy	84.55	82.27	91.82	10.45	7.27	5.91	5.00	10.45	2.27	
	Herbs like adaptogens	87.27	78.64	90.91	5.91	12.27	5.91	6.82	9.09	3.18	
	Yoga, breathing, etc.	68.18	54.09	65.91	19.55	21.36	21.36	12.27	24.55	12.73	

Table S3. Outer Loadings Results.

Latent variables	Manifest variable	Weight		
		New Zealand	India	Malaysia
Attitudes (ATT)	I think drinking stress-reducing drinks in the future is enjoyable (ATT1)<br>I think drinking stress-reducing drinks will be a pleasant sensory experience (ATT2)<br>I think it is important to drink stress-reducing drinks in the future (ATT3)<br>It is beneficial to drink stress-reducing drinks in the future to improve my health (ATT4)<br>I think consuming stress-reducing drinks will help me to face stressful situations (ATT5)	0.785<br>0.822<br>0.841<br>0.844<br>0.732	0.843<br>0.855<br>0.905<br>0.928<br>0.875	0.809<br>0.831<br>0.891<br>0.819<br>0.799
Subjective Norms (SN)	My friends and family agree I should consume stress-reducing drinks (SN1)<br>My family and friends think and feel positively when I consume stress-reducing drinks (SN2)<br>My friends and family will buy and consume stress-reducing drinks (SN3)	0.864<br>0.883<br>0.847	0.868<br>0.926<br>0.886	0.871<br>0.848<br>0.840
Perceived Behavioural Control Items (PBC)	I will drink stress-reducing drinks completely by myself without any external influences from family and friends (PBC1)<br>I will decide entirely for myself to drink stress-reducing drinks (PBC2)<br>I feel empowered when I buy stress-reducing drinks (PBC3)	0.867<br>0.848<br>0.790	0.836<br>0.823<br>0.792	0.858<br>0.798<br>0.827

	The decision to buy stress-reducing drinks will be completely up to myself (PBC4)	0.614	0.736	0.688
Behavioural Intention Items (BI)	I will for sure drink stress-reducing drinks in the future (BI1)	0.891	0.901	0.918
	How often do you plan to drink stress-reducing drinks?(BI2)	0.664	0.468	0.472
	I would also recommend others to buy and consume stress-reducing drinks (BI3)	0.898	0.885	0.905
Functional Value (FV)	Stress-reducing drinks improve health and reduce stress (FV1)	0.805	0.881	0.813
	Stress-reducing drinks are healthier than energy drinks (FV2)	0.752	0.892	0.766
	Stress-reducing drinks are free from food additives (FV3)	0.806	0.903	0.767
	Stress-reducing drinks are strong in product safety (FV4)	0.815	0.867	0.806
Social Value (SV)	When I consume stress-reducing drinks I feel admired by other people (SV1)	0.870	0.906	0.909
	I feel accepted by others when I buy stress-reducing drinks (SV2)	0.938	0.928	0.936
	I feel respected by others when buying stress-reducing drinks (SV3)	0.936	0.930	0.941
	Consuming stress-reducing drinks support me in how people see and assess me (SV4)	0.886	0.913	0.899
Conditional Value (CV)	I decide to purchase stress-reducing drinks when they are offered with discounts or with promotional offers (CV1)	0.789	0.856	0.769
	I consume stress-reducing drinks instead of conventional drinks like energy drinks under aggravating environmental circumstances (CV2)	0.885	0.910	0.849
	I prefer consuming stress-reducing drinks instead of conventional drinks like energy drinks under subsidiary conditions (CV3)	0.875	0.889	0.813
Epistemic Value (EV)	I search for information about stress-reducing drinks before buying them (EV1)	0.853	0.849	0.872
	I prefer to gain information about stress-reducing drinks and compare all available options of stress-reducing drinks before I decide to acquire them (EV2)	0.882	0.868	0.911
	Consuming stress-reducing drinks will be a new adventure for me (EV3)	0.550	0.865	0.776
Initial Trust (IT)	I believe that stress-reducing drinks work to reduce stress (IT1)	0.861	0.908	0.854
	I believe stress-reducing drinks are trustworthy (IT2)	0.933	0.936	0.902
	I believe stress-reducing drinks are authentic (IT3)	0.818	0.928	0.856

Table S4. Results of Discriminant Validity.

Country		SN	PBC	ATT	FV	IT	SV	CV	EV	BI
New Zealand	SN	**1**	0.319	0.343	0.353	0.217	0.223	0.219	0.116	0.421
	PBC	0.319	1	0.437	0.312	0.201	0.148	0.306	0.119	0.439
	ATT	0.343	0.437	1	0.280	0.195	0.187	0.263	0.139	0.415
	FV	0.353	0.312	0.280	1	0.399	0.185	0.358	0.187	0.377
	IT	0.217	0.201	0.195	0.399	1	0.303	0.344	0.216	0.283
	SV	0.223	0.148	0.187	0.185	0.303	1	0.270	0.094	0.213

	CV	0.219	0.306	0.263	0.358	0.344	0.270	1	0.302	0.390
	EV	0.116	0.119	0.139	0.187	0.216	0.094	0.302	1	0.196
	BI	0.421	0.439	0.415	0.377	0.283	0.213	0.390	0.196	1
India	SN	1	0.165	0.168	0.220	0.160	0.263	0.109	0.023	0.342
	PBC	0.165	1	0.410	0.342	0.487	0.160	0.320	0.324	0.420
	ATT	0.168	0.410	1	0.337	0.457	0.143	0.338	0.263	0.471
	FV	0.220	0.342	0.337	1	0.609	0.215	0.323	0.315	0.531
	IT	0.160	0.487	0.457	0.609	1	0.209	0.456	0.516	0.472
	SV	0.263	0.160	0.143	0.215	0.209	1	0.179	0.067	0.268
	CV	0.109	0.320	0.338	0.323	0.456	0.179	1	0.320	0.315
	EV	0.023	0.324	0.263	0.315	0.516	0.067	0.320	1	0.198
	BI	0.342	0.420	0.471	0.531	0.472	0.268	0.315	0.198	1
Malaysia	SN	1	0.329	0.317	0.196	0.276	0.066	0.212	0.190	0.261
	PBC	0.329	1	0.371	0.266	0.289	0.069	0.130	0.153	0.432
	ATT	0.317	0.371	1	0.208	0.277	0.099	0.140	0.067	0.340
	FV	0.196	0.266	0.208	1	0.614	0.180	0.308	0.177	0.373
	IT	0.276	0.289	0.277	0.614	1	0.245	0.334	0.227	0.430
	SV	0.066	0.069	0.099	0.180	0.245	1	0.173	0.055	0.167
	CV	0.212	0.130	0.140	0.308	0.334	0.173	1	0.306	0.302
	EV	0.190	0.153	0.067	0.177	0.227	0.055	0.306	1	0.147
	BI	0.261	0.432	0.340	0.373	0.430	0.167	0.302	0.147	1

Table S5. Detailed Measurement Invariance Assessment (MICOM) Results by Construct and Country Pair

Construct	Pair	Step 2: c	Step 2: p	Comp. Inv.	Step 3a: Mean Diff.	Step 3a: p	Mean Eq.	Step 3b: Var. Diff.	Step 3b: p	Var. Eq.	Overall
Attitude	NZ–India	0.999	.134	Yes	-0.139	.074	Yes	-0.195	.088	Yes	Full
Attitude	NZ–Malaysia	0.999	.136	Yes	-0.172	.035	No	0.231	.068	Yes	Partial
Attitude	India–Malaysia	1.000	.541	Yes	-0.016	.433	Yes	0.430	.003	No	Partial

Construct	Pair	Step 2: c	Step 2: p	Comp. Inv.	Step 3a: Mean Diff.	Step 3a: p	Mean Eq.	Step 3b: Var. Diff.	Step 3b: p	Var. Eq.	Overall
Behavioural Intention	NZ–India	0.997	.117	Yes	-0.079	.203	Yes	0.220	.051	Yes	Full
Behavioural Intention	NZ–Malaysia	0.998	.086	Yes	-0.294	.001	No	0.281	.020	No	Partial
Behavioural Intention	India–Malaysia	1.000	.755	Yes	-0.239	.006	No	0.070	.314	Yes	Partial
Conditional Value	NZ–India	1.000	.614	Yes	-0.075	.218	Yes	-0.111	.197	Yes	Full
Conditional Value	NZ–Malaysia	0.999	.507	Yes	-0.325	.000	No	0.481	.000	No	Partial
Conditional Value	India–Malaysia	1.000	.655	Yes	-0.240	.006	No	0.595	.000	No	Partial
Epistemic Value	NZ–India	0.979	.030	No	-0.341	.000	—	-0.018	.445	—	Not established
Epistemic Value	NZ–Malaysia	0.991	.213	Yes	-0.539	.000	No	0.245	.029	No	Partial
Epistemic Value	India–Malaysia	0.998	.404	Yes	-0.168	.033	No	0.273	.041	No	Partial
Functional Value	NZ–India	1.000	.565	Yes	-0.137	.078	Yes	-0.303	.026	No	Partial
Functional Value	NZ–Malaysia	0.999	.732	Yes	-0.050	.300	Yes	0.096	.299	Yes	Full
Functional Value	India–Malaysia	0.999	.204	Yes	0.087	.183	Yes	0.404	.006	No	Partial
Initial Trust	NZ–India	1.000	.241	Yes	-0.261	.003	No	-0.242	.054	Yes	Partial
Initial Trust	NZ–Malaysia	1.000	.803	Yes	-0.165	.041	No	0.172	.156	Yes	Partial
Initial Trust	India–Malaysia	0.999	.112	Yes	0.119	.109	Yes	0.411	.001	No	Partial
Perceived Behavioural Control	NZ–India	0.997	.227	Yes	-0.156	.049	No	0.031	.407	Yes	Partial
Perceived Behavioural Control	NZ–Malaysia	0.998	.345	Yes	-0.253	.005	No	0.341	.015	No	Partial

Construct	Pair	Step 2: c	Step 2: p	Comp. Inv.	Step 3a: Mean Diff.	Step 3a: p	Mean Eq.	Step 3b: Var. Diff.	Step 3b: p	Var. Eq.	Overall
Perceived Behavioural Control	India–Malaysia	0.998	.462	Yes	-0.088	.177	Yes	0.311	.028	No	Partial
Subjective Norms	NZ–India	0.999	.120	Yes	0.332	.000	No	-0.038	.386	Yes	Partial
Subjective Norms	NZ–Malaysia	1.000	.736	Yes	-0.110	.125	Yes	0.307	.018	No	Partial
Subjective Norms	India–Malaysia	0.999	.225	Yes	-0.453	.000	No	0.338	.007	No	Partial
Social Value	NZ–India	0.999	.153	Yes	-0.016	.440	Yes	-0.179	.070	Yes	Full
Social Value	NZ–Malaysia	1.000	.860	Yes	0.006	.467	Yes	0.119	.184	Yes	Full
Social Value	India–Malaysia	0.999	.078	Yes	0.019	.425	Yes	0.297	.006	No	Partial

Table S6. Discriminant validity assessed using the heterotrait–monotrait ratio (HTMT) for the TPB and TCV constructs across New Zealand, India and Malaysia.

**Construct pair**	**New Zealand**	**India**	**Malaysia**
Behavioural intention ↔ Attitudes	0.781	0.806	0.801
Conditional value ↔ Attitudes	0.615	0.649	0.437
Conditional value ↔ Behavioural intention	0.785	0.714	0.728
Epistemic value ↔ Attitudes	0.487	0.586	0.460
Epistemic value ↔ Behavioural intention	0.607	0.537	0.459
Epistemic value ↔ Conditional value	0.761	0.661	0.718
Functional value ↔ Attitudes	0.613	0.622	0.570
Functional value ↔ Behavioural intention	0.738	**0.881**	0.742

Construct pair	New Zealand	India	Malaysia
Functional value ↔ Conditional value	0.732	0.638	0.710
Functional value ↔ Epistemic value	0.610	0.650	0.493
Initial trust ↔ Attitudes	0.522	0.732	0.602
Initial trust ↔ Behavioural intention	0.649	0.824	0.806
Initial trust ↔ Conditional value	0.708	0.761	0.728
Initial trust ↔ Epistemic value	0.646	0.815	0.564
Initial trust ↔ Functional value	0.754	**0.852**	**0.951**
Perceived behavioural control ↔ Attitudes	0.783	0.709	0.630
Perceived behavioural control ↔ Behavioural intention	0.787	0.776	0.593
Perceived behavioural control ↔ Conditional value	0.676	0.660	0.566
Perceived behavioural control ↔ Epistemic value	0.479	0.707	0.552
Perceived behavioural control ↔ Functional value	0.675	0.648	0.482
Perceived behavioural control ↔ Initial trust	0.535	0.788	0.575
Subjective norms ↔ Attitudes	0.686	0.439	0.697
Subjective norms ↔ Behavioural intention	0.796	0.744	0.766
Subjective norms ↔ Conditional value	0.572	0.381	0.474
Subjective norms ↔ Epistemic value	0.458	0.165	0.315
Subjective norms ↔ Functional value	0.712	0.519	0.542
Subjective norms ↔ Initial trust	0.556	0.445	0.632

Construct pair	New Zealand	India	Malaysia
Subjective norms ↔ Perceived behavioural control	0.664	0.430	0.614
Social value ↔ Attitudes	0.481	0.389	0.273
Social value ↔ Behavioural intention	0.546	0.628	0.478
Social value ↔ Conditional value	0.599	0.467	0.497
Social value ↔ Epistemic value	0.410	0.276	0.264
Social value ↔ Functional value	0.490	0.499	0.500
Social value ↔ Initial trust	0.627	0.492	0.561
Social value ↔ Perceived behavioural control	0.405	0.388	0.235
Social value ↔ Subjective norms	0.538	0.560	0.357

Table S7. Covariate-adjusted sensitivity analysis: effects of demographic variables on behavioural intention in the New Zealand, Indian and Malaysian samples.

	**New Zealand**			**India**			**Malaysia**		
**Covariate → Behavioural intention**	***β***	***p***	**95% CI**	***β***	***p***	**95% CI**	***β***	***p***	**95% CI**
Age	−0.040	0.514	[−0.167, 0.073]	0.069	0.160	[−0.017, 0.154]	−0.014	0.839	[−0.151, 0.107]
Gender	−0.026	0.551	[−0.115, 0.065]	0.003	0.933	[−0.072, 0.077]	0.016	0.723	[−0.090, 0.109]
Ethnicity	0.061	0.184	[−0.029, 0.163]	**−0.090**	**0.022**	**[−0.186, −0.012]**	0.016	0.719	[−0.083, 0.100]
Income	0.029	0.546	[−0.054, 0.134]	−0.061	0.154	[−0.138, 0.027]	−0.041	0.418	[−0.146, 0.070]

Covariate → Behavioural intention	New Zealand			India			Malaysia		
	β	*p*	95% CI	*β*	*p*	95% CI	*β*	*p*	95% CI
Educational qualification	0.019	0.686	[−0.053, 0.104]	0.005	0.904	[−0.064, 0.079]	**0.115**	**0.026**	**[0.010, 0.240]**
Marital status	0.035	0.495	[−0.042, 0.135]	−0.036	0.475	[−0.135, 0.066]	0.023	0.698	[−0.071, 0.122]
Household composition	−0.050	0.331	[−0.160, 0.058]	0.004	0.927	[−0.077, 0.081]	0.025	0.599	[−0.080, 0.134]
Household size	0.031	0.533	[−0.073, 0.139]	−0.002	0.970	[−0.090, 0.079]	−0.028	0.546	[−0.119, 0.057]

Table S8. Sensitivity analysis of the behavioural intention (BI) composite: structural path estimates with and without item BI2.

Path	With BI2 (BI1, BI2, BI3)			Without BI2 (BI1, BI3)			
	β	*p*	95% CI	*β*	*p*	95% CI	Δβ
New Zealand							
SN → BI	0.239	<0.001	[0.107, 0.337]	0.247	<0.001	[0.088, 0.365]	+0.008
PBC → BI	0.216	0.001	[0.075, 0.379]	0.233	<0.001	[0.083, 0.398]	+0.017
ATT → BI	0.170	0.005	[0.042, 0.272]	0.135	0.027	[0.003, 0.277]	−0.035
FV → BI	0.089	0.164	[−0.097, 0.259]	0.126	**0.049**	[−0.016, 0.262]	+0.037
SV → BI	0.012	0.820	[−0.109, 0.124]	−0.007	0.904	[−0.107, 0.109]	−0.019
CV → BI	0.186	0.004	[0.034, 0.360]	0.159	0.013	**[−0.002, 0.323]**	−0.027

	With BI2 (BI1, BI2, BI3)			Without BI2 (BI1, BI3)			
**Path**	***β***	***p***	**95% CI**	***β***	***p***	**95% CI**	**Δβ**
EV → BI	0.055	0.282	[−0.035, 0.163]	0.072	0.166	[−0.043, 0.184]	+0.017
IT → BI	0.051	0.403	[−0.101, 0.227]	0.049	0.424	[−0.082, 0.186]	−0.002
**R^2 (BI)**		0.636			0.633		ΔR^2 −0.003
India							
SN → BI	0.192	<0.001	[0.096, 0.308]	0.187	<0.001	[0.104, 0.285]	−0.005
PBC → BI	0.159	0.005	[0.031, 0.310]	0.139	0.015	**[−0.003, 0.277]**	−0.020
ATT → BI	0.261	<0.001	[0.143, 0.402]	0.280	<0.001	[0.155, 0.422]	+0.019
FV → BI	0.338	<0.001	[0.231, 0.506]	0.328	<0.001	[0.189, 0.466]	−0.010
SV → BI	0.080	0.090	[−0.022, 0.179]	0.081	0.088	[−0.010, 0.165]	+0.001
CV → BI	0.040	0.456	[−0.077, 0.153]	0.009	0.865	[−0.071, 0.099]	−0.031
EV → BI	−0.078	0.173	[−0.196, 0.068]	−0.022	0.707	[−0.127, 0.095]	+0.056
IT → BI	0.051	0.530	[−0.176, 0.187]	0.049	0.545	[−0.140, 0.204]	−0.002
**R^2 (BI)**		0.705			0.701		ΔR^2 −0.004
Malaysia							
SN → BI	0.146	0.015	[0.051, 0.301]	0.131	0.030	[0.016, 0.269]	−0.015

	With BI2 (BI1, BI2, BI3)			Without BI2 (BI1, BI3)			
**Path**	***β***	***p***	**95% CI**	***β***	***p***	**95% CI**	**Δβ**
PBC → BI	−0.004	0.950	[−0.125, 0.159]	−0.016	0.788	[−0.170, 0.169]	−0.012
ATT → BI	0.345	<0.001	[0.089, 0.573]	0.334	<0.001	[0.059, 0.566]	−0.011
FV → BI	0.095	0.186	[−0.081, 0.276]	0.125	0.083	[−0.073, 0.364]	+0.030
SV → BI	0.061	0.225	[−0.048, 0.202]	0.064	0.208	[−0.038, 0.178]	+0.003
CV → BI	0.209	0.001	[0.099, 0.343]	0.194	0.002	[0.036, 0.312]	−0.015
EV → BI	−0.049	0.374	[−0.167, 0.055]	−0.014	0.794	[−0.121, 0.092]	+0.035
IT → BI	0.193	0.014	[0.029, 0.398]	0.183	0.021	[0.011, 0.343]	−0.010
**R^2 (BI)**	0.616			0.609			ΔR^2 −0.007
